# 3*d*_z_2 orbital delocalization and magnetic collapse in superconducting (La,Pr)_3_Ni_2_O_7-δ_ films

**DOI:** 10.1093/nsr/nwag518

**Published:** 2026-08-25

**Authors:** Xiaoyang Chen, Wenliang Zhang, Fei Peng, Ting Cui, Guangdi Zhou, Zezhong Li, Jaewon Choi, Lizhi Xu, Yiu-Fung Chiu, Stefano Agrestini, Sahil Tippireddy, Haoliang Huang, Heng Wang, Xianfeng Wu, Peng Li, Jin-Feng Jia, Mirian Garcia-Fernandez, Yi Lu, Er-Jia Guo, Qi-Kun Xue, Zhuoyu Chen, Donglai Feng, Ke-Jin Zhou

**Affiliations:** Diamond Light Source, Harwell Campus, Didcot OX11 0DE, UK; National Synchrotron Radiation Laboratory and School of Nuclear Science and Technology, University of Science and Technology of China, Hefei 230026, China; State Key Laboratory of Quantum Functional Materials, Department of Physics, Guangdong Provincial Key Laboratory of Topological Matter, and Guangdong Basic Research Center of Excellence for Quantum Science, Southern University of Science and Technology, Shenzhen 518055, China; Beijing National Laboratory for Condensed Matter Physics and Institute of Physics, Chinese Academy of Sciences, Beijing 100190, China; Department of Physics & Center of Materials Science and Optoelectronics Engineering, University of Chinese Academy of Sciences, Beijing 100049, China; State Key Laboratory of Quantum Functional Materials, Department of Physics, Guangdong Provincial Key Laboratory of Topological Matter, and Guangdong Basic Research Center of Excellence for Quantum Science, Southern University of Science and Technology, Shenzhen 518055, China; Quantum Science Center of Guangdong-Hong Kong-Macao Greater Bay Area, Shenzhen 518045, China; National Synchrotron Radiation Laboratory and School of Nuclear Science and Technology, University of Science and Technology of China, Hefei 230026, China; Diamond Light Source, Harwell Campus, Didcot OX11 0DE, UK; State Key Laboratory of Quantum Functional Materials, Department of Physics, Guangdong Provincial Key Laboratory of Topological Matter, and Guangdong Basic Research Center of Excellence for Quantum Science, Southern University of Science and Technology, Shenzhen 518055, China; Diamond Light Source, Harwell Campus, Didcot OX11 0DE, UK; Department of Physics, University of Oxford, Clarendon Laboratory, Parks Road, Oxford OX1 3PU, UK; Diamond Light Source, Harwell Campus, Didcot OX11 0DE, UK; Diamond Light Source, Harwell Campus, Didcot OX11 0DE, UK; State Key Laboratory of Quantum Functional Materials, Department of Physics, Guangdong Provincial Key Laboratory of Topological Matter, and Guangdong Basic Research Center of Excellence for Quantum Science, Southern University of Science and Technology, Shenzhen 518055, China; Quantum Science Center of Guangdong-Hong Kong-Macao Greater Bay Area, Shenzhen 518045, China; State Key Laboratory of Quantum Functional Materials, Department of Physics, Guangdong Provincial Key Laboratory of Topological Matter, and Guangdong Basic Research Center of Excellence for Quantum Science, Southern University of Science and Technology, Shenzhen 518055, China; Quantum Science Center of Guangdong-Hong Kong-Macao Greater Bay Area, Shenzhen 518045, China; State Key Laboratory of Quantum Functional Materials, Department of Physics, Guangdong Provincial Key Laboratory of Topological Matter, and Guangdong Basic Research Center of Excellence for Quantum Science, Southern University of Science and Technology, Shenzhen 518055, China; State Key Laboratory of Quantum Functional Materials, Department of Physics, Guangdong Provincial Key Laboratory of Topological Matter, and Guangdong Basic Research Center of Excellence for Quantum Science, Southern University of Science and Technology, Shenzhen 518055, China; Quantum Science Center of Guangdong-Hong Kong-Macao Greater Bay Area, Shenzhen 518045, China; State Key Laboratory of Quantum Functional Materials, Department of Physics, Guangdong Provincial Key Laboratory of Topological Matter, and Guangdong Basic Research Center of Excellence for Quantum Science, Southern University of Science and Technology, Shenzhen 518055, China; Quantum Science Center of Guangdong-Hong Kong-Macao Greater Bay Area, Shenzhen 518045, China; Diamond Light Source, Harwell Campus, Didcot OX11 0DE, UK; National Laboratory of Solid State Microstructures and Department of Physics, Nanjing University, Nanjing 210093, China; Collaborative Innovation Center of Advanced Microstructures, Nanjing University, Nanjing 210093, China; Beijing National Laboratory for Condensed Matter Physics and Institute of Physics, Chinese Academy of Sciences, Beijing 100190, China; Department of Physics & Center of Materials Science and Optoelectronics Engineering, University of Chinese Academy of Sciences, Beijing 100049, China; State Key Laboratory of Quantum Functional Materials, Department of Physics, Guangdong Provincial Key Laboratory of Topological Matter, and Guangdong Basic Research Center of Excellence for Quantum Science, Southern University of Science and Technology, Shenzhen 518055, China; Quantum Science Center of Guangdong-Hong Kong-Macao Greater Bay Area, Shenzhen 518045, China; State Key Laboratory of Quantum Functional Materials, Department of Physics, Guangdong Provincial Key Laboratory of Topological Matter, and Guangdong Basic Research Center of Excellence for Quantum Science, Southern University of Science and Technology, Shenzhen 518055, China; Quantum Science Center of Guangdong-Hong Kong-Macao Greater Bay Area, Shenzhen 518045, China; New Cornerstone Laboratory, Hefei National Laboratory, Hefei 230088, China; National Synchrotron Radiation Laboratory and School of Nuclear Science and Technology, University of Science and Technology of China, Hefei 230026, China

**Keywords:** nickelate superconductors, orbital delocalization, spin–density–wave, resonant inelastic X-ray scattering (RIXS)

## Abstract

The recent discovery of Ruddlesden–Popper (RP) nickelate thin-film superconductors has opened a new frontier in unconventional superconductivity. Its realization requires both compressive epitaxial strain and highly oxidative growth conditions, yet the microscopic pathway from the parent phase to the superconducting phase remains elusive. Here, X-ray absorption spectra and resonant inelastic X-ray scattering are employed to track this evolution by independently tuning strain and oxygen content in (La,Pr)$_3$Ni$_2$O$_{7-\delta }$ thin films. We uncover a remarkable two-step narrative. First, signatures of delocalization emerge: spectral weight transfers from an ‘Upper Hubbard’-like peak to the hole-like peak associated with O $2p_z$ state, and in parallel, the initially localized Ni $3d_{z^2}$ orbital becomes more itinerant followed by the broadening and weakening of *d–d* orbital excitations. Second, as itinerancy increases, long-range spin–density–wave (SDW) order is suppressed in both intensity and correlation length, indicating direct competition with superconductivity. Yet, short-range magnons persist: they become damped but their bandwidth stays unchanged. Our results paint a coherent picture that both strain and oxygenation drive the RP bilayer nickelates toward the superconducting instability, where the O $2p_z$ and Ni $3d_{z^2}$ orbitals become delocalized. Concomitantly, the long-range magnetic order loses coherence and gets suppressed. These findings establish an orbital-selective route to RP nickelate superconductivity, in which the emergence and progressive delocalization of the interlayer $3d_{z^2}$–$2p_z$–$3d_{z^2}$ channel and the robust short-range magnons upon the melting of SDW order are prerequisites, providing strong constraints for theory and the roadmap for designing nickelate superconductors.

## Teaser

The study reveals how compressive strain and oxygen content reshape the electronic structure and magnetism of (La,Pr)3Ni2O7-d films, uncovering an orbital-selective pathway from the non-superconducting state towards superconductivity.

## INTRODUCTION

The Ruddlesden–Popper (RP) nickelates ($R_{n+1}\mathrm{Ni}_n\mathrm{O}_{3n+1}$) have emerged as a new family of high-temperature superconductors. Structurally, they consist of *n* layers of perovskite-like $\mathrm{NiO}_6$ octahedra stacked along the *c* axis and separated by rock-salt $\mathrm{LaO}$ layers, with adjacent octahedra sharing apical oxygens. Superconductivity is realized under hydrostatic pressure, with the superconducting transition temperature ($T_\mathrm{c}$) tuned by the dimensionality (i.e., the layer number *n*), or, the stacking sequence [1–6]. Among this family, the Sm-doped bilayer RP nickelate exhibits the highest reported $T_\mathrm{c}$, reaching *∼ 96* K under pressure [6]. In contrast to cuprate superconductors, both Ni $d_{x^2-y^2}$ and $d_{z^2}$ orbitals contribute to

the low-energy electronic structure [7,8], giving rise to multi-orbital characteristics reminiscent of iron-based superconductors [9]. A variety of theoretical models have been proposed to describe its electronic structure and the pairing symmetry under pressure, with ongoing debate regarding the relative importance of the partially filled Ni $d_{z^2}$ and $d_{x^2-y^2}$ orbitals in driving superconductivity [10]. Besides, the evolution of the magnetic properties such as the spin–density–wave (SDW) is critical to identify the pairing mechanism [11]. However, direct experimental verification remains challenging due to the extreme conditions imposed by high pressure.

Recent advances in thin-film growth techniques have enabled the synthesis of RP and hybrid RP nickelate films on various substrates, introducing tunable biaxial strain via lattice mismatch. Superconductivity has been realized in compressively strained (La,Pr)$_3$Ni$_2$O$_{7-\delta }$ films and related superstructures, with $T_\mathrm{c,onset}$ reaching 60 K on SrLaAlO$_4$ (SLAO, *∼ -2*% strain) and 12 K on LaAlO$_3$ (LAO, *∼ -1*% strain) [12–18], providing an accessible route to investigate the superconducting mechanism at ambient pressure. Structurally, compressive strain reduces the in-plane lattice constant and suppresses the tilting of the $\mathrm{NiO}_6$ octahedra, driving the system toward a higher-symmetry configuration [19].

Besides the structural change, the strain also leads to modifications in the electronic and magnetic properties. Density functional theory (DFT) calculations predict that the $d_{z^2}$-derived bands shift away from the Fermi level ($E_\mathrm{F}$), though under hydrostatic pressure they may cross $E_\mathrm{F}$ [20–22]. Experimentally, it has been an important question for angle-resolved photoemission spectroscopy (ARPES) studies regarding the position and role of the $d_{z^2}$-derived bands in compressively strained films [18,23–27]. Concerning the spin degree of freedom, the interlayer exchange coupling ($J_z$) is predicted to be enhanced owing to the reduced tilting of NiO$_6$ octahedra under compressive strain, which may play a key role in stabilizing superconductivity, a scenario supported by recent resonant inelastic X-ray scattering (RIXS) studies [28]. Optical spectroscopy revealed the evolution of a density-wave-like gap in La$_3$Ni$_2$O$_{7-\delta }$ under pressure, which suggests a potential competition between density–wave order and superconductivity [29]. However, the direct experimental probe of such density–wave order evolution in compressively strained thin films remains unexplored.

From a materials perspective, oxygen deficiency has been identified as a critical challenge for superconductivity in RP bilayer nickelates [15,30]. These vacancies are detrimental to superconductivity because they may destroy the Ni 3$d_{z^2}$ interlayer coupling and in-plane phase coherence [31–33]. Beyond vacancies, interstitial oxygen atoms by extra oxygenation may induce hole carriers that suppress superconductivity [33,34]. It is thus pivotal to experimentally explore the impact of oxygen content on the electronic and magnetic properties.

In this work, we track the microscopic evolution in (La,Pr)$_3$Ni$_2$O$_{7-\delta }$ thin films from a non-superconducting parent phase to a superconducting phase using X-ray absorption spectra (XAS) and resonant inelastic X-ray scattering, where compressive strain and oxygen content serve as two parallel tuning knobs. Extracting from the O *K*-XAS data, we find that the O $2p_z$ and the Ni $3d_{z^2}$ orbitals become more delocalized upon tuning of the strain or oxygen content. The Ni *L*-XAS and RIXS results exhibit that the XAS excitation and *d–d* orbital excitations associated with Ni $3d_{z^2}$ orbital character become broadened and less pronounced despite a rigid crystal-field splitting upon the two independent tunings. Accompanying this orbital-selective delocalization, long-range spin–density–wave order is suppressed in both intensity and correlation length, in a direct competition with superconductivity. Short-range magnons become damped but retain their bandwidth, challenging predictions of enhanced interlayer exchange coupling. Our results establish an orbital-selective route to superconductivity in (La,Pr)$_3$Ni$_2$O$_{7-\delta }$ for which the emergence and progressive delocalization of the interlayer $3d_{z^2}$–$2p_z$–$3d_{z^2}$ channel and the robust short-range magnons are prerequisites providing strong constraints for theoretical studies and designing nickelate superconductors.

## RESULTS

### Engineering of (La,Pr)$_3$Ni$_2$O$_{7-\delta }$ thin films

We designed two series of thin films to investigate the effects of compressive strain and oxygen stoichiometry independently (see growth details in Method). The strain-tuning series includes two non-superconducting La$_3$Ni$_2$O$_{7-\delta }$ thin films grown on (001)-oriented LaAlO$_3$ and SrLaAlO$_4$ substrates (non-SC LNO/LAO and non-SC LNO/SLAO1), which impose biaxial in-plane compressive strains of approximately *-1%* and *-2%*, respectively (Fig. 1a). These films are identical to those reported in Ref. [35]. They were grown under the same oxygen partial pressure; therefore viewed as containing a comparable oxygen content. Both samples exhibit insulating behavior at low temperatures. The epitaxial strain is effectively transferred to the thin films, leading to systematic structural modifications [35]. Furthermore, the $\mathrm{NiO}_6$ octahedra become more straightened compared to the La$_3$Ni$_2$O$_7$ bulk single crystal (LNO bulk) at ambient pressure (Note S1).

**Figure 1.**
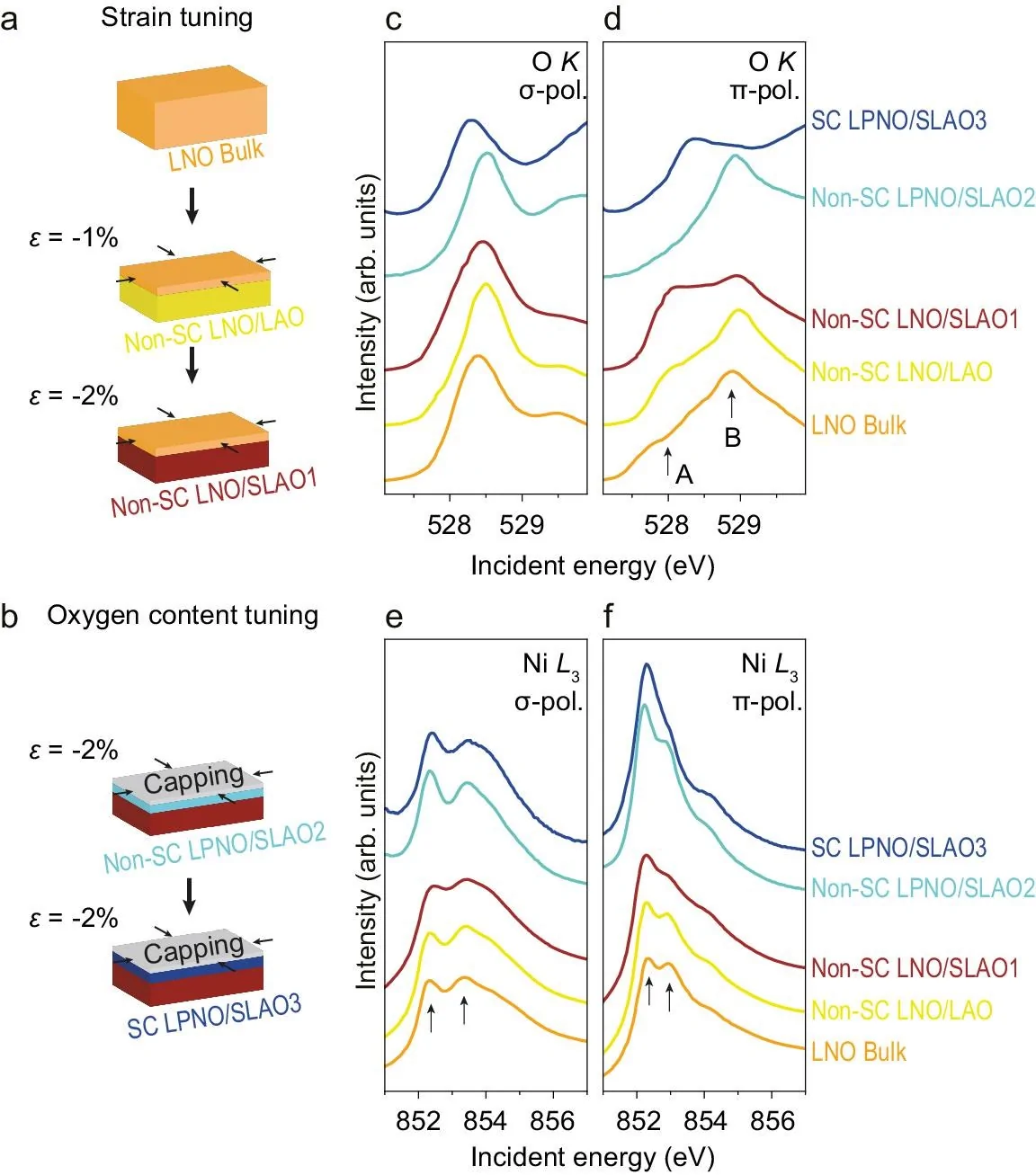
The O *K*- and Ni $L_3$- X-ray absorption spectra. (a and b) Schematic illustration of (La,Pr)$_3$Ni$_2$O$_{7-\delta }$ films designed with varying compressive strain (a) and oxygen content (b). The LPNO/SLAO3 is superconducting at low temperature, while the other samples are insulating. (c and d) O *K*-XAS measured at σ polarization (c) and *σ* polarization (d). (e and f) Ni $L_3$-XAS measured at σ polarization (e) and *σ* polarization (f). XAS were measured in total fluorescence yield (TFY) mode at 20 K with a grazing incidence angle of 20°, where *σ* polarization predominantly probes out-of-plane orbital states, while σ polarization probes in-plane orbital states.

The oxygen content tuning series includes two (La,Pr)$_3$Ni$_2$O$_{7-\delta }$ thin films grown on the SrLaAlO$_4$ substrate [13,16,36]. Both samples were grown under an ozone–oxygen atmosphere and capped with a SrTiO$_3$ layer [37] (Fig. 1b). Oxygen content is controlled *in situ*, such that one sample is insulating at low temperatures (non-SC LPNO/SLAO2), whereas the other shows a $T_\mathrm{c,onset}$ at approximately 50 K (SC LPNO/SLAO3) (Note S2).

In the following sections, we discuss the XAS and RIXS results to track the evolution of the electronic and magnetic properties of (La,Pr)$_3$Ni$_2$O$_{7-\delta }$ under the two independent tuning routes, that is, the compressive strain and the oxygen content.

### Electronic excitations

Figure 1c and d shows the linear polarization-dependent Oxygen *K*-edge X-ray absorption (XAS) spectra from the thin films and LNO bulk reference (See XAS experiments in Methods and the experimental geometry in Note S3). The O *K*-XAS is a very powerful tool for probing the orbital-dependent unoccupied density of states above the Fermi level [38–40]. For the in-plane direction (σ-pol., $E \mathbin {//} a$), a single-peak profile dominates the pre-edge dictating the O $2p_{x,y}$ orbital character. No appreciable difference is observed in the strain-dependent films in comparison to the LNO bulk. The oxygen content dependent films feature the same pre-edge profile as the LNO bulk, although the resonance of the SC LPNO/SLAO3 has a slight red-shift by *∼ 0.2* eV. This is due to the slightly higher hole-doping level in the SC LPNO/SLAO3 compared to the oxygen-deficient non-SC LPNO/SLAO2. The out-of-plane pre-edge (*σ*-pol., $E \mathbin {//} c$), characterized by a two-peak structure, manifests a much greater evolution where the spectral weight transfers from the second peak (peak B) to the first peak (peak A) in both sample series, particularly the oxygen content dependence. The high-energy peak is related to the ‘upper Hubbard band’ of a rather undoped material; the low-energy peak stems from doping-induced oxygen ligand holes. In our (La,Pr)$_3$Ni$_2$O$_{7-\delta }$ thin films, the spectral weight transfer from the ‘upper Hubbard band’-like peak B to the hole-like peak A is reminiscent of the evolution of the O *K*-XAS pre-edge in La$_{2-x}$Sr$_x$NiO$_4$ and La$_{2-x}$Sr$_x$CuO$_4$ as a function of Sr doping [38–40]. The same trend in the strain- and the oxygen content series unambiguously unveils that the two tuning methods work effectively and drive La$_3$Ni$_2$O$_{7-\delta }$ from a Mott-like insulating state toward a more itinerant superconducting state. In contrast to La$_{2-x}$Sr$_x$NiO$_4$ where the spectral weight gain of the hole states is realized in both O $2p_{x,y}$ and $2p_{z}$ orbitals; here, in (La,Pr)$_3$Ni$_2$O$_{7-\delta ,}$ it predominantly happens in the O $2p_{z}$ orbital states suggesting that the apical oxygen is much more susceptible upon tuning underscoring its critical role in superconductivity.

Figure 1e and f shows the Ni $L_3$-edge XAS spectra from the thin films and the LNO bulk. The Ni $L_3$ XAS spectra of the LNO bulk exhibit characteristic peaks at *∼*852.3 and *∼*853.3 eV for σ-pol (with the latter at *∼ *853.0 eV for *σ*-pol), corresponding to the main $2p^53d^8$ and the satellite $2p^53d^8\underline{L}$ configuration, respectively [8]. Here, $\underline{L}$ denotes the O *2p* ligand hole. The two-peak XAS profile is persistent in both series with little change in the excitation to the Ni $3d_{x^2-y^2}$ states (σ-pol). For the excitation to the Ni $3d_{z^2}$ states (*σ*-pol), the satellite feature becomes progressively broadened and less pronounced as a function of the strain and oxygen content tuning, namely, from LNO/LAO to LNO/SLAO1, and from LPNO/SLAO2 to SC LPNO/SLAO3.

In Fig. 2a and b, the incident-energy dependent Ni $L_3$-RIXS spectra illustrate the overall profile of Ni *3d* orbital excitations. Similar to the evolution of the satellite peak in the Ni $L_3$-XAS, the Ni *d–d* orbital excitations retain mostly their energy positions under the current energy resolution (*∼*40 meV) while becoming gradually broadened and weakened, particularly ${dd}_2$ at *∼*1.6 eV and $dd_3$ at *∼*0.4 eV (Fig. 2c–g), in both film series. Known from the RIXS study of the LNO bulk, ${dd}_3$ at *∼*0.4 eV is sensitive to the interlayer $3d_{z^2}$–$2p_z$–$3d_{z^2}$ hopping [8]. The much more pronounced changes in the electronic structure of the O *2p* orbitals than in the Ni *3d* orbitals are reminiscent of a typical charge-transfer Mott system.

**Figure 2.**
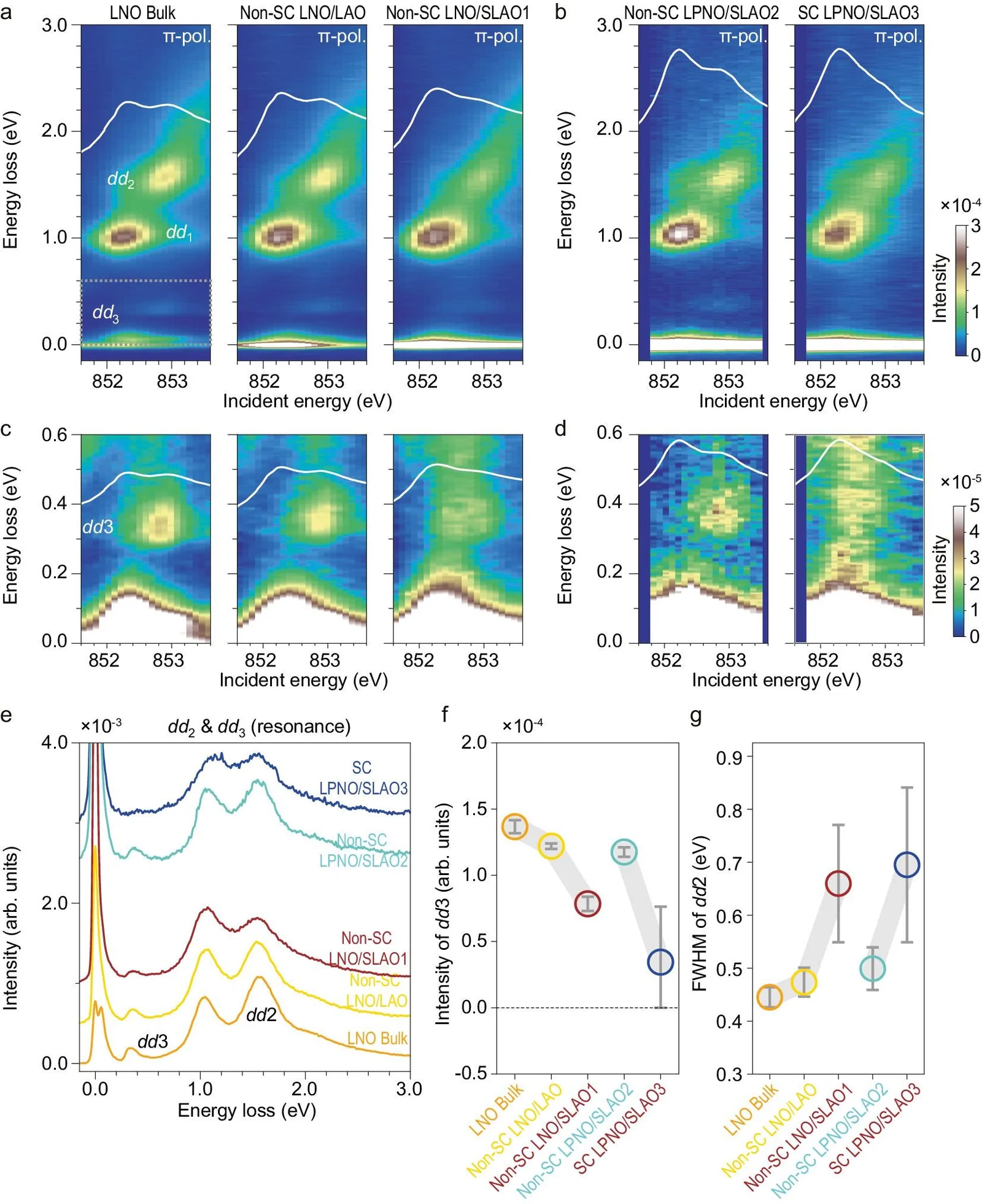
Electronic excitations. (a and b) TFY XAS and energy-dependent RIXS maps at the Ni $L_3$ edge. The RIXS spectra are normalized by the high-energy *d–d* excitations with integrated intensity over the energy-loss window [0.6 eV, 3.0 eV]. Both XAS and RIXS were measured at a grazing incidence angle of 20° with *σ* polarization to enhance sensitivity to out-of-plane $d_{z^2}$ orbital states. The RIXS map of LNO bulk is adapted from Ref. [8]. (c and d) Zoomed-in views of the RIXS maps shown in (a and b), highlighting the $dd_3$ excitations. (e) Integrated RIXS intensity over the incident-energy range [852.5 eV, 853.3 eV], corresponding to the resonance of the $dd_2$ and $dd_3$ excitations. (f) Intensity of the $dd_3$ excitation across bulk and different thin films. (g) FWHM of the $dd_2$ excitation across LNO bulk and thin films. Both $dd_2$ and $dd_3$ features are fitted using Lorentzian functions, with error bars representing the standard deviations of the fitting parameters.

Generally, a well-defined XAS peak or a *d–d* orbital excitation is indicative of the Mott phase of a strongly correlated system; contrarily, a broadened or featureless XAS or RIXS excitation usually results from a rather delocalized system [41]. The negligible changes in excitation energies indicate that the local crystal field is only weakly affected by the tuning. The spectral weight transfer and the broadening illustrated in the spectroscopic data highlight that the out-of-plane orbitals become delocalized under the drive of the compressive strain and the oxygenation from the insulating state towards the superconducting state.

### Magnetic excitations

Figure 3a shows the momentum-dependent magnetic excitations in LNO/LAO along the two high-symmetry directions (see Methods). The damped harmonic oscillator (DHO) function was used to fit the magnons. The extracted magnon peaks ($\omega _p$) exhibit a dispersion overlapping with that of LNO bulk at ambient pressure (Fig. 3b) [8]. For a detailed comparison, representative RIXS spectra at $\mathbf {q}_\parallel = (-0.14, -0.14)$ (Fig. 3d) and $\mathbf {q}_\parallel = (-0.06, -0.06)$ (Fig. 3e) are presented. In contrast to the LNO bulk, the magnons in the thin films are significantly damped. The extracted damping factor (*γ*) in the thin film is significantly broader than that of the LNO bulk. In the more strongly strained non-SC LNO/SLAO1 sample, the magnon presents nearly the same dispersion as that of the LNO bulk and the thin film LNO/LAO (Fig. 3f). The extracted damping factor differs little between the LNO/SLAO1 and the LNO/LAO (Fig. 3b and c).

**Figure 3.**
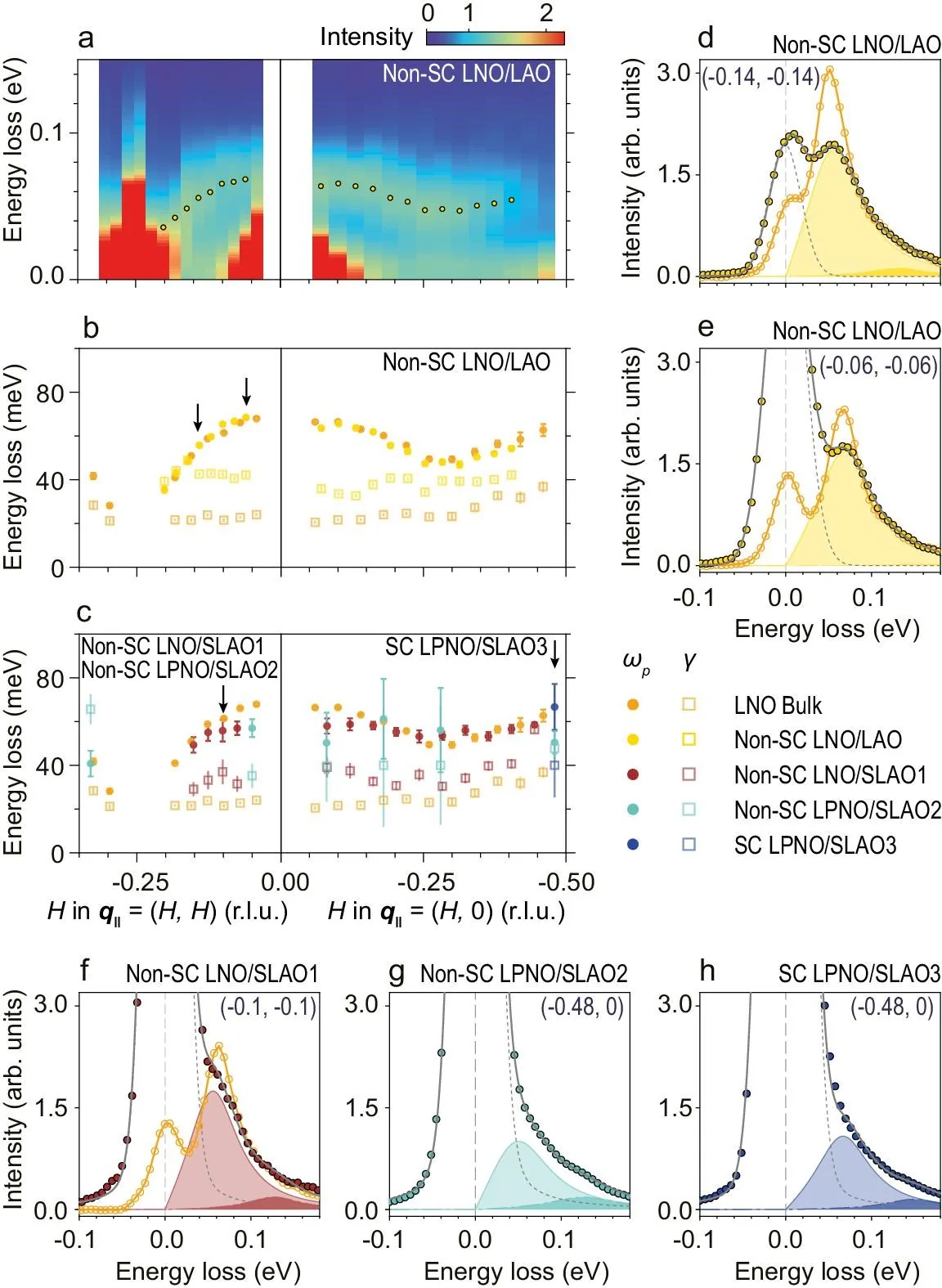
Magnetic excitations. (a) Momentum-resolved RIXS intensity maps of the LNO/LAO film along the ${\mathbf{q}}_\parallel =(H,H)$ and *(H,0)* directions. The Brillouin zone is defined based on the pseudo-tetragonal lattice [8]. All spectra were measured using *σ*-polarized X-rays tuned to the magnon resonance (852.4 eV). The magnon excitations were fitted by the damped harmonic oscillator (DHO) function. The circle markers denote the extracted magnon peak energies ($\omega _p$), with error bars representing the fitting uncertainties, which are smaller than the marker size (Note S4). At ${\mathbf{q}}_\parallel \approx 0$, strong elastic scattering arises from the specular geometry; these data are excluded from the colormap. (b and c) Magnon dispersion ($\omega _p$ vs ${\mathbf{q}}_\parallel$) for LNO/LAO, LNO/SLAO1, LPNO/SLAO2, and LPNO/SLAO3. The $\omega _p$ of LNO bulk are overlaid for comparison, which were processed from data in Ref. [8]. The damping factors (*γ*) are shown as semi-transparent squares. (d–h) Representative RIXS spectra and fittings at ${\mathbf{q}}_\parallel$ points indicated by arrows in (b and c). The magnon spectral weight is highlighted by colored shading, and the dashed line denotes the elastic peak. In panels (d–f), RIXS spectra of LNO bulk [8] at the same ${\mathbf{q}}_\parallel$ point (orange markers) are overlaid for comparison and their intensities were normalized to the high-energy *d–d* excitations.

For the oxygen content dependent thin film series, non-SC LPNO/SLAO2 and SC LPNO/SLAO3, despite the dominant elastic signal in RIXS spectra owing to the unit-cell-thick films, magnons are recognizable as an asymmetrical tail to the elastic peak (Fig. 3g and h). Using the DHO fitting method, the magnetic excitation and the damping factor at several momenta are extracted, as shown in Fig. 3c. Within the instrumental uncertainty, the tuning of oxygen content does not bring any sizable impact on the magnon bandwidth and damping. Given the limitations of the strong elastic peak, the precise magnon dispersion in the non-SC LPNO/SLAO2 and SC LPNO/SLAO3 requires more detailed studies, such as employing 90° scattering angle where the elastic peak can be strongly suppressed [42].

Compared to the well-defined magnons in the LNO bulk which is approximately a strongly correlated system, the much-damped magnons in the thin films, nearing a more itinerant electron system, portray a paramagnon with reduced lifetime and coherence under either the compressive strain or the oxygen content tuning, driving ultimately towards the superconducting phase. The latter is much like the persistent but damped paramagnons well established in cuprate superconductors [43].

### Spin–density–wave

At low temperature, LNO bulk exhibits a long-range SDW order, characterized by a prominent scattering peak at the in-plane wave vector $\mathbf {q}_{\parallel }^{\mathrm{SDW}}$ = *(0.25, 0.25)* (Fig. 4a) [8]. In the compressively strained LNO/LAO and LNO/SLAO1, an SDW peak is also observed, albeit at a slightly reduced in-plane wave vector (Fig. 4b and c). Interestingly, the SDW intensity is progressively suppressed upon the compressive strain tuning (Fig. 4a–c). Its correlation length (*ξ*) also decreases gradually, from *∼*30 nm in the bulk crystal, to *∼*13 nm and *∼*10 nm in LNO/LAO and LNO/SLAO1, respectively (Fig. 4h). Furthermore, both the SDW intensity and the correlation length manifest clear temperature dependence: they weaken gradually at elevated temperatures and become barely discernible above *∼*150 K (Fig. 4f and g). In the non-SC LPNO/SLAO2, the SDW is also present at a slightly shifted wave vector, with an intensity and correlation length comparable to those observed in the strain-tuned films (Fig. 4d). This shift of SDW wave vector may reflect an intrinsic modification of the Fermi surface induced by compressive strain and is worthy of further investigation. Remarkably, following a thorough investigation, no SDW order was revealed in the SC LPNO/SLAO3 film (Fig. 4e) (Note S5). Since superconductivity was ensured before and after the RIXS measurement, the non-discernible SDW signal indicates that the long-range magnetic order competes with superconductivity and is fully suppressed in the superconducting state.

**Figure 4.**
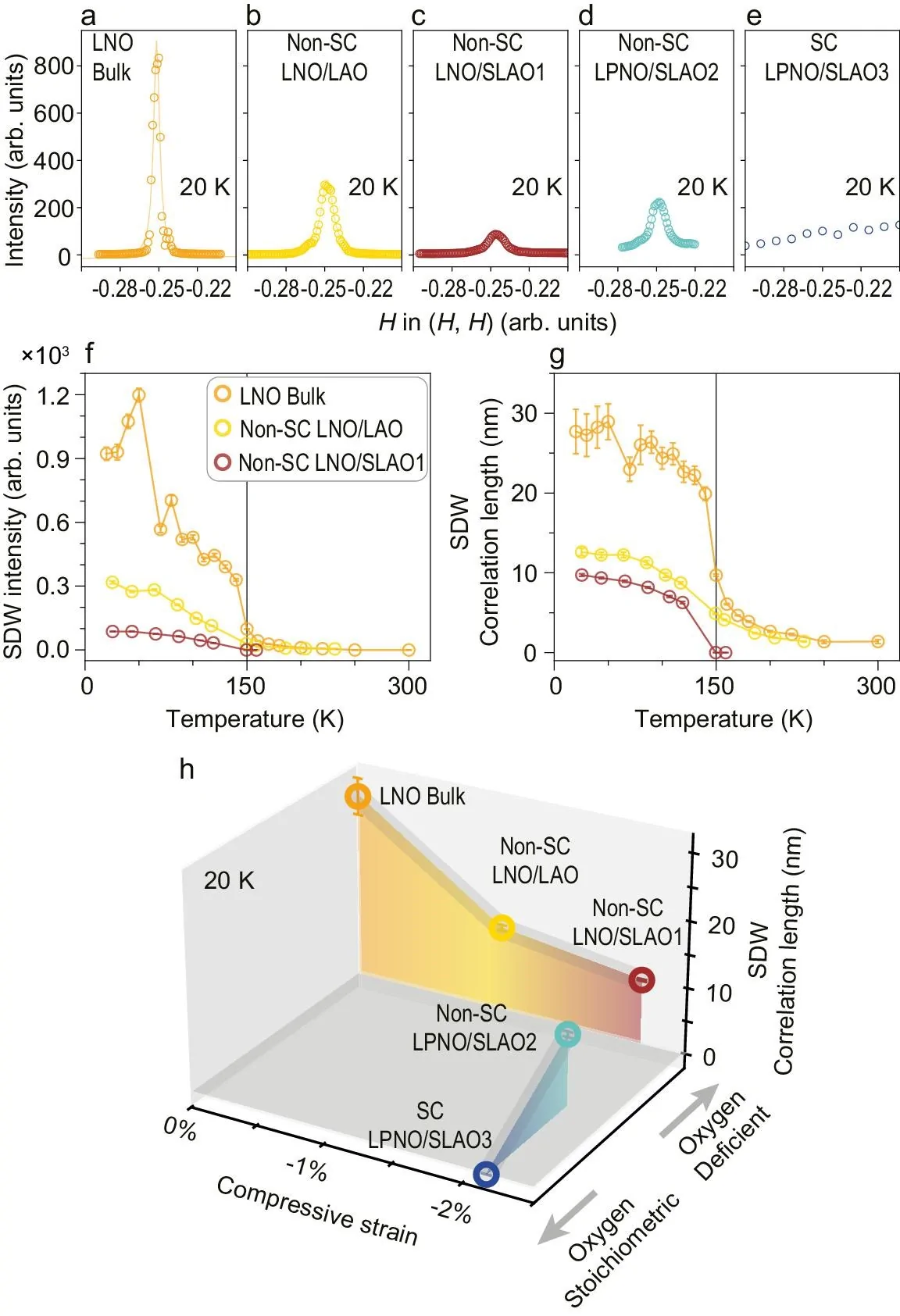
Spin–density–wave. (a–e) SDW peaks of LNO bulk and thin films. RIXS spectra were measured along *(H,H)* near the SDW wave vector *(-0.25,-0.25)* at 20 K using *σ*-polarized X-rays tuned to 852.4 eV. The spectra were normalized to the 1 eV *d–d* excitations for quantitative comparison between different samples. The SDW peak was integrated over [−0.02, 0.02] eV and fitted with a Lorentzian. (f and g) Temperature dependence of the SDW intensity (f) and correlation length ($\xi _{\mathrm{SDW}} = 1/\mathrm{HWHM}$) (g), where HWHM is the half-width at half-maximum of the SDW peak. $T_\mathrm{SDW} = 150$ K for the LNO bulk is indicated. (h) Summary of the $\xi _{\mathrm{SDW}}$ as a function of compressive strain and oxygen content for the bulk and thin film samples at 20 K. Data for the LNO bulk were extracted and processed from Ref. [8].

If the LNO bulk is viewed as the ‘‘undoped’’ parent compound, the oxygen-deficient non-SC LPNO/SLAO2 films can be regarded as ‘‘underdoped’’ LNO in which the oxygen deficiency directly alters the carrier concentration acting as chemical doping (filling-controlled). In the strained non-SC LNO/LAO and LNO/SLAO1, although the epitaxial strain does not introduce extra carriers, it primarily modifies the lattice geometry and corresponding electronic structure (bandwidth-controlled), exhibiting phenomenological similarities to the underdoped samples. Here, the reduced SDW intensity and correlation length are signatures of competition between magnetism and superconductivity instability before it gets fully suppressed in the near ‘optimally’ doped SC LPNO/SLAO3 sample (Fig. 4h). Dissecting the phase-diagram like picture informs us that the oxygen vacancies are detrimental to superconductivity [44–47], and, the eventual realization of bulk superconductivity in La$_3$Ni$_2$O$_{7-\delta }$ films requires both the compressive strain and the macroscopic oxygen stoichiometry [12,13,16,17].

## DISCUSSION

The combination of X-ray absorption spectroscopy and the resonant inelastic X-ray scattering unfolded a rich landscape of the electronic structure and the magnetic properties of the (La,Pr)$_3$Ni$_2$O$_{7-\delta }$ thin films under the two parallel tuning routes that is, the compressive strain and the oxygen content. We firstly learned from the O *K*-XAS that the spectral weight transfer from the upper Hubbard band like peak to the O $2p_z$ hole state is realized upon tuning. The broadening effect encoded in the Ni *L*-XAS and RIXS collectively confirms that the tuning drives the delocalization of the correlated $d_{z^2}$ state. Both the spectral weight transfer and the delocalization are well-known spectroscopic fingerprints imprinted in the hole-doped single-layer cuprates and nickelate evolving from an antiferromagnetic insulator to a more delocalized and metallic system [39,48]. It is remarkable to see that the above spectroscopic fingerprints are concerted in the two independent tuning routes in (La,Pr)$_3$Ni$_2$O$_7$ when driving toward the superconducting state.

Uncovering the development of the electronic structure of the RP nickelates from the non-superconducting to the superconducting phase is one of the salient results of our study, which can provide a strong constraint for future theoretical studies. Importantly, the predominant involvement of the O $p_z$ and the Ni $3d_{z^2}$ orbitals in the process lends to a coherent picture: the O $p_z$ and the Ni $3d_{z^2}$ orbitals, initially localized under the zero strain or oxygen deficient state, become delocalized via the two tuning methods; gradually the interlayer $3d_{z^2}$–$2p_z$–$3d_{z^2}$ orbital channel emerges and becomes progressively delocalized and coherent before getting involved in superconductivity. It is worth noting that the energy position of the $d_{z^2}$-derived *γ* band remains under active debate, with conflicting conclusions reported by recent ARPES studies [18,23–27]. As soft X-ray RIXS and XAS are considerably more bulk sensitive and much less affected by surface conditions, our results provide an important complementary perspective on the evolution of the $d_{z^2}$ electronic states. The negligible involvement of the Ni $3d_{x^2-y^2}$ orbital in the tuning process is consistent with the fact that it is already itinerant prior to forming the Cooper pairing, as evidenced by the Fermi surface topology [18,23–27,49]. Also, the fixed *d–d* orbital excitation energies as well as the near-constant absorption thresholds of the O *K*- and Ni $L_3$- edges allude to a robust crystal-field splitting with respect to the Fermi level. Our result differs from the DFT calculations, which suggest a substantial shift of the $d_{z^2}$-dominated band away from the Fermi level (by several hundred meV) [22,50].

The short-range spin fluctuation is regarded as one of the potential mechanisms for the unconventional superconductivity. Previously, the dispersive magnon bandwidth in La$_3$Ni$_2$O$_7$ was reported to increase under the compressively strained films [28]. Our RIXS results reveal that the magnon becomes increasingly damped upon tuning either the strain or the oxygen content, whereas its bandwidth or magnetic energy scale changes only slightly (see details in Note S4). The enhanced magnon damping may primarily originate from the increased in-plane itinerancy induced by the substrate-imposed compressive strain. The robust magnon bandwidth, which is dominated by $J_z$, suggests that the corresponding exchange interaction is largely retained upon the tuning of the strain and the oxygen content. However, our result is in stark contrast to the theoretical proposal of a significant enhancement of $J_z$ by more than 50% in the superconducting phase compared to ambient pressure [21]. Despite the robust short-range magnetic fluctuation, the long-range SDW order shows a strong reduction in both ordering intensity and the correlation length upon the tuning of the strain and the oxygen content, establishing its direct competition with superconductivity consistent with the findings by optical spectroscopy [29]. The result advocates a close link between RP nickelates and other unconventional high-temperature superconductors.

Lastly, we note that the structural inhomogeneity and quenched disorder are likely introduced in strained films. However, our STEM data show that the epitaxial strain is transferred from the substrate to the films with good crystallinity (Fig. S1). In addition, the unchanged *d–d* orbital excitation at *∼*1 eV between the LNO bulk and the thin films highlights that the electronic structure is largely unaffected by the structural inhomogeneity or the quenched disorder, if any.

In summary, the study of (La,Pr)$_3$Ni$_2$O$_{7-\delta }$ thin films reveals that compressive strain and oxygenation collectively drive the delocalization of the $3d_{z^2}$ and $2p_z$ orbitals and the suppression of long-range spin–density–wave order, which together induce the high-temperature superconducting state. This scenario is evidenced by three spectroscopic signatures: (1) spectral weight transfer in XAS, (2) broadening of the *d–d* excitations, and (3) damping of magnon excitations. These findings establish the essential prerequisites for superconductivity in the Ruddlesden–Popper nickelates, paving a definitive path for the design of novel superconductors.

*Note added:* Upon completion of this work, we became aware of an independent RIXS study on superconducting (La,Pr)$_3$Ni$_2$O$_7$ thin films [42].

## METHODS

### Growth of thin films

For the strain tuning thin films (non-superconducting LNO/LAO and LNO/SLAO1), high-quality La$_3$Ni$_2$O$_{7-\delta }$ thin films were deposited on (001)-oriented LaAlO$_3$ and SrLaAlO$_4$ substrates by pulsed laser deposition (PLD), with a uniform thickness of approximately 30 nm. A stoichiometric La$_3$Ni$_2$O$_7$ polycrystalline target was synthesized via a solid-state reaction route employing La$_2$O$_3$ and NiO powders. Prior to deposition, the base pressure was maintained at 1.33 *×* 10$^{-8}$ mbar. During the growth process, the oxygen partial pressure and substrate temperature were set at 4 *×* 10$^{-1}$ mbar and 750°C, respectively. The energy density was 1.25 J cm$^{-2}$, and the laser frequency was maintained at 5 Hz. All films underwent post-annealing in an oxygen-rich atmosphere ($P_{\mathrm{O}_2}$ = 1.33 *×* 10$^2$ mbar), followed by slow cooling to room temperature. The structural properties of the films were characterized using X-ray diffraction techniques, including 2*θ*-*ω* scans, rocking curves, and reciprocal space mapping (RSM), with a Panalytical X’Pert3 MRD diffractometer equipped with a Cu $K_{\alpha 1}$ source. The film thickness was determined through X-ray reflectivity (XRR) and analyzed using GenX software. Cross-sectional transmission electron microscopy (TEM) specimens, exhibiting various strain states, were prepared via the focused ion beam (FIB) lift-off process. High-angle annular dark-field scanning transmission electron microscopy (HAADF-STEM) and annular bright-field scanning transmission electron microscopy (ABF-STEM) experiments were conducted using a JEM ARM 200CF microscope at the Institute of Physics (IOP), Chinese Academy of Sciences. The resistivity curves (*ρ*–*T*) and RSM data are presented in Ref. [35]. Under non-experimental conditions, the samples were stored at room temperature in a rough-vacuum desiccator.

For the oxygen-content-tuned thin films (non-superconducting LPNO/SLAO2 and superconducting LPNO/SLAO3), high-quality (La,Pr)$_3$Ni$_2$O$_{7-\delta }$ films were grown by the gigantic-oxidative atomic-layer-by-layer epitaxy (GAE) method [16,36], in which (La,Pr)O$_x$ and NiO$_x$ targets were alternately ablated using a pulsed laser. The (La,Pr)O$_x$ target has a nominal composition of (La$_{0.65}$Pr$_{0.35}$)O. This alternating deposition sequence produces a stacking structure of (La,Pr)O–NiO$_2$–(La,Pr)O–NiO$_2$–(La,Pr)O, resulting in a nominal composition of La$_{1.95}$Pr$_{1.05}$Ni$_2$O$_{7-\delta }$. On as-received SrLaAlO$_4$ substrates (MTI-Kejing), a (La,Pr)O–NiO$_2$–(La,Pr)O buffer layer was first deposited to mitigate interfacial structural discontinuities. During growth, layer-by-layer control was achieved by synergistically tuning the laser pulse energy and pulse sequence, together with real-time monitoring of the reflection high-energy electron diffraction intensity oscillations. No post-annealing treatment was performed for any of the samples. In both the SC-LPNO/SLAO3 and non-SC LPNO/SLAO2 samples, the (La,Pr)$_3$Ni$_2$O$_{7-\delta }$ layer was grown with a thickness of three unit cells. For the STO capping layer, an 800-pulse deposition yielded an estimated thickness of *∼*4.4 nm on the LPNO/SLAO3 sample, and a 400-pulse deposition resulted in *∼*2.2 nm on the LPNO/SLAO2 sample.

The non-SC LPNO/SLAO2 and SC LPNO/SLAO3 were grown at a substrate temperature of 850°C, measured from the back side of the Inconel flag-type sample holder. For SC LPNO/SLAO3, the laser pulse ratio was 101:148 under an ozone partial pressure of $1.04\times 10^{-2}$ mbar and a total oxygen pressure of $1.15\times 10^{-1}$ mbar. After film growth, the sample was first cooled at 100°C min^−1^ to below 200°C under the same oxidative environment. Subsequently, an SrTiO3 capping layer was deposited at room temperature using 800 laser pulses at 8 Hz and 600 mJ laser energy under the same growth atmosphere. For non-SC LPNO/SLAO2, the laser pulse ratio was set to 107:138 (pulses on (La,Pr)O$_x$ : NiO$_x$). The film was grown in a mixed ozone/oxygen atmosphere with an ozone partial pressure of $1.40\times 10^{-2}$ mbar and a total oxygen pressure of $1.15\times 10^{-1}$ mbar. After deposition, a SrTiO$_3$ capping layer was grown *in situ* using 400 laser pulses at 4 Hz with a laser energy of 600 mJ under the same oxidative conditions while maintaining the substrate temperature at 850°C. The sample was then cooled at a rate of 100°C min^−1^ to below 200°C in the same atmosphere. To minimize potential oxygen loss, LPNO/SLAO2 and LPNO/SLAO3 were transferred in a refrigerated container and maintained below −13°C for *∼*24 h during transport to Diamond Light Source. Upon arrival, the samples were rapidly transferred and kept at *20*–$60\, \mathrm{K}$ for RIXS experiments. The samples were transported back to China using the same protocol, with temperatures maintained below −20°C throughout transit.

### XAS and RIXS measurements

Experiments were performed at Beamline I21 at Diamond Light Source [51]. In this work, the structural properties of the La$_3$Ni$_2$O$_7$ single crystal and (La,Pr)$_3$Ni$_2$O$_{7-\delta }$ thin films are described using a pseudo-tetragonal unit cell with lattice parameters $a^T$, $b^T$, and *c*. For the La$_3$Ni$_2$O$_7$ single crystal, reciprocal lattice units (r.l.u.) are defined based on the lattice parameters $a^T = b^T \approx 3.833$ Å and *c = 20.45* Å. Accordingly, with $2\sigma /a^T = 2\sigma /b^T = 2\sigma /c = 1$, the momentum transfer is expressed as $\mathbf {Q} = H\mathbf {a}^{T*} + K\mathbf {b}^{T*} + L\mathbf {c}^{*}$, where *H, K*, and *L* are given in reciprocal lattice units [8]. For the (La,Pr)$_3$Ni$_2$O$_{7-\delta }$ thin films, the in-plane lattice parameters are defined by the corresponding substrates. Specifically, for LNO/LAO, $a^T = b^T \approx 3.792$ Å, while for LNO/SLAO or LPNO/SLAO, $a^T = b^T \approx 3.750$ Å. The crystallographic $a^T$–*c* ($b^T$–*c*) plane of the sample was aligned within the horizontal scattering plane. Sample’s polar angular offsets (*θ* and *χ*) and azimuthal offset (*ϕ*) were aligned by the SDW peak, such that the $c^{*}$ axis lies in the scattering plane. The spectrometer arm was at a fixed position of *Ω =*154°. XAS spectra were collected with a grazing incidence angle of $\theta _0 = $20° to probe both in-plane and out-of-plane unoccupied states. All XAS measurements were done at a temperature of 20 K with the exit slit opening to 30 *μ*m. Total fluorescence yield XAS spectra were collected and normalized to the incoming beam intensity. Both linear vertical (σ) and horizontal (*σ*) polarizations were used. Energy-dependent RIXS measurements were performed at the grazing incidence angle of $\theta _0 = $20° and the temperature of 20 K. The exit slit was open to 30 *μ*m corresponding to an average energy resolution of 42 meV (FWHM). The incident energy range went from 851.6 to 853.6 eV in steps of 0.2 eV (or 0.15 eV) to fully capture the resonance behaviour across the Ni-$L_3$ absorption peaks. Momentum-dependent RIXS measurements were performed at the resonant energy of 852.4 eV at a temperature of 20 K with the exit slit opening to 20 *μ*m corresponding to an average energy resolution of 39 meV (FWHM). The momentum resolution is 0.002 r.l.u. near the SDW wave vector at the Ni $L_3$-edge. RIXS spectra were collected using both *π* polarizations. The grazing incidence geometry ($\theta < \Omega /2$) was applied for the acquisition of RIXS spectra shown in the main text.

## Supplementary Material

nwag518_Supplemental_File
