# Supplementary material for: 3dz2 orbital delocalization and magnetic collapse in superconducting (La,Pr)3Ni2O7-δ films
Source: Natl Sci Rev. 2026 Aug 25;13(19):nwag518. doi: 10.1093/nsr/nwag518 (PMC13725838; doi:10.1093/nsr/nwag518)
Supplement: Supplementary file 1 [file nwag518_supplemental_file.pdf]

Supplementary Information for
"$3d_{z^2}$ orbital delocalization and magnetic collapse in superconducting $(La,Pr)_3Ni_2O_{7-\delta}$ films"

Xiaoyang Chen,[1,†] Wenliang Zhang,[2,†] Fei Peng,[3,†] Ting Cui,[4,5,†] Guangdi Zhou,[3,6,†] Zezhong Li,[2] Jaewon Choi,[1] Lizhi Xu,[3] Yiu-Fung Chiu,[1,7] Stefano Agrestini,[1] Sahil Tippireddy,[1] Haoliang Huang,[3,6] Heng Wang,[3,6] Xianfeng Wu,[3] Peng Li,[3,6] Jin-Feng Jia,[3,6] Mirian Garcia-Fernandez,[1] Yi Lu,[8,9] Er-Jia Guo,[4,5,*] Qi-Kun Xue,[3,6] Zhuoyu Chen,[3,6,*] Donglai Feng,[10,*] and Ke-Jin Zhou[2,*]

[1]*Diamond Light Source, Harwell Campus, Didcot OX11 0DE, United Kingdom*
[2]*National Synchrotron Radiation Laboratory and School of Nuclear Science and Technology, University of Science and Technology of China, Hefei 230026, China*
[3]*State Key Laboratory of Quantum Functional Materials, Department of Physics, Guangdong Provincial Key Laboratory of Topological Matter, and Guangdong Basic Research Center of Excellence for Quantum Science, Southern University of Science and Technology, Shenzhen 518055, China*
[4]*Beijing National Laboratory for Condensed Matter Physics and Institute of Physics, Chinese Academy of Sciences, Beijing, 100190, China*
[5]*Department of Physics & Center of Materials Science and Optoelectronics Engineering, University of Chinese Academy of Sciences, Beijing, 100049, China*
[6]*Quantum Science Center of Guangdong-Hong Kong-Macao Greater Bay Area, Shenzhen, 518045, China*
[7]*Department of Physics, University of Oxford, Clarendon Laboratory, Parks Road, Oxford OX1 3PU, United Kingdom*
[8]*National Laboratory of Solid State Microstructures and Department of Physics, Nanjing University, Nanjing 210093, China*
[9]*Collaborative Innovation Center of Advanced Microstructures, Nanjing University, Nanjing 210093, China*
[10]*New Cornerstone Laboratory, Hefei National Laboratory, Hefei 230088, China*

Supplementary Note 1. The strain dependence of the out-of-plane Ni-O-Ni bond angle.

For the strain-tuning samples LNO/LAO and LNO/SLAO1, epitaxial strain is efficiently transferred from the substrate to the nickelate layers, producing compressed in-plane and elongated out-of-plane lattice parameters [S1]. Beyond lattice distortion, epitaxial strain also strongly reshapes the Ni–O–Ni bond angle along the bilayer c axis (φ_c; Fig. S1a). Figure S1b summarizes

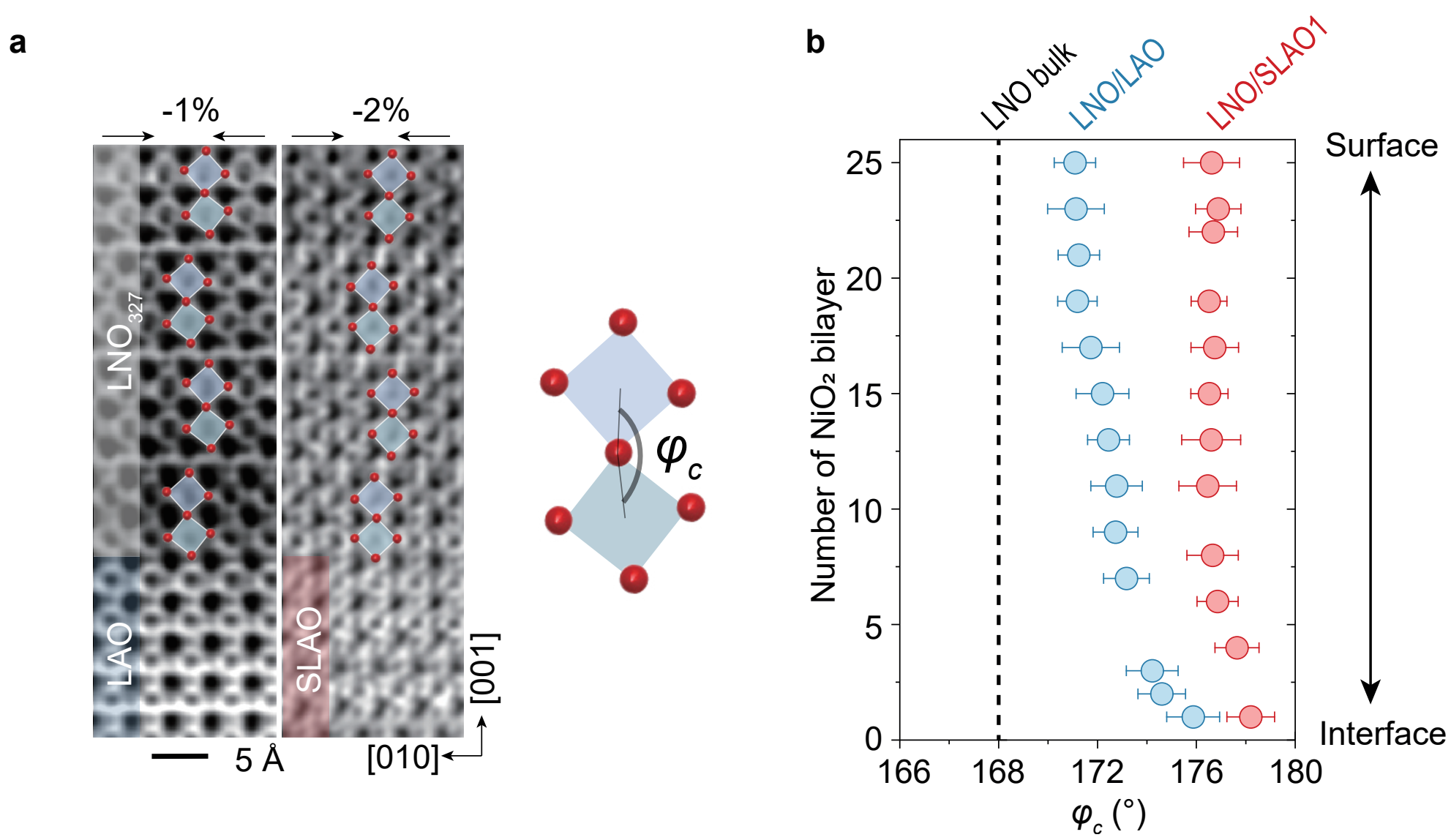

FIG. S1. **Out-of-plane Ni–O–Ni bond angle in strain-tuning samples. (a)** Atomically resolved scanning transmission electron microscopy (STEM) images of LNO/LAO and LNO/SLAO1. The in-plane lattice mismatch between bulk LNO and the substrates is indicated. The out-of-plane Ni–O–Ni bond angle (φ_c) is defined schematically. **(b)** Spatial distribution of φ_c across the film thickness. The bulk value (φ_c = 168°) is marked by the dashed line.

† These authors contributed equally to this work.
* Corresponding authors. E-mails: ejguo@iphy.ac.cn; chenzhuoyu@sustech.edu.cn; dlfeng@ustc.edu.cn; kjzhou@ustc.edu.cn.

φ_c across the film thickness. Although both films show a modest reduction in φ_c from interface to surface, compressive strain progressively straightens the NiO_6 octahedra, with the effect most pronounced in the more compressively strained LNO/SLAO1. Comparable strain-driven tuning of both lattice parameters and φ_c has also been reported in Ref. [S2].

Although a structural gradient may exist, the near-surface region remains under compressive strain, with the strain increasing from LNO bulk to LNO/LAO and LNO/SLAO1. Since the probing depth of soft X-ray XAS/RIXS at the Ni L edge is on the order of the film thickness (~ 30 nm) even in a grazing-incidence geometry, the measured spectra represent the average response of the entire film rather than the topmost relaxed layers. Therefore, any surface relaxation has only a limited influence on the measured electronic and magnetic excitations or on the comparison between different strain states.

Supplementary Note 2. The electric transport of $(La,Pr)_3Ni_2O_{7-\delta}$ films before and after RIXS experiments.

Figure S2a shows the temperature-dependent resistivity of the oxygen-content tuning samples LPNO/SLAO2 and LPNO/SLAO3 prior to the RIXS experiments. LPNO/SLAO2 exhibits insulating behavior at low temperatures, whereas LPNO/SLAO3 shows a superconducting transition upon cooling with a critical temperature T_c^{onset} at ~ 50 K. It is worth noting that a two-step superconducting transition is observed, a feature commonly seen in RP bilayer nickelate thin films. Previous studies have shown that oxygen loss can occur at both the in-plane and apical oxygen sites [S3], and even a minute amount of oxygen deficiency can disrupt the global phase coherence within the Ni–O planes. This manifests as a lower temperature at which zero resistance is achieved [S4]. Importantly, such subtle oxygen loss has a negligible impact on the pairing strength [S5], which is fundamentally distinct from the substantial oxygen deficiency that drives the insulating behavior discussed in our work. Therefore, the two-step transition does not affect our scientific conclusions.

To perform RIXS experiments, the sample was transported from Shenzhen to the UK under pure oxygen encapsulation at −50 °C, which minimized oxygen loss. (see Method in the main text). After the RIXS experiments, we confirmed that the superconductivity of LPNO/SLAO3 is preserved (Fig. S2b). The return trip was conducted at room temperature without encapsulation, resulting in slight oxygen deficiency. Consistently, the two-step transition becomes slightly more pronounced in the post-RIXS transport data (Fig. S2b), further supporting this scenario. It is important to emphasize that zero resistance was unambiguously observed both before and after the RIXS experiments, confirming that the sample remained fully superconducting throughout the synchrotron measurements. Achieving this is technically highly challenging and has so far been realized by only a very limited number of research teams worldwide, further underscoring the robustness of our conclusions.

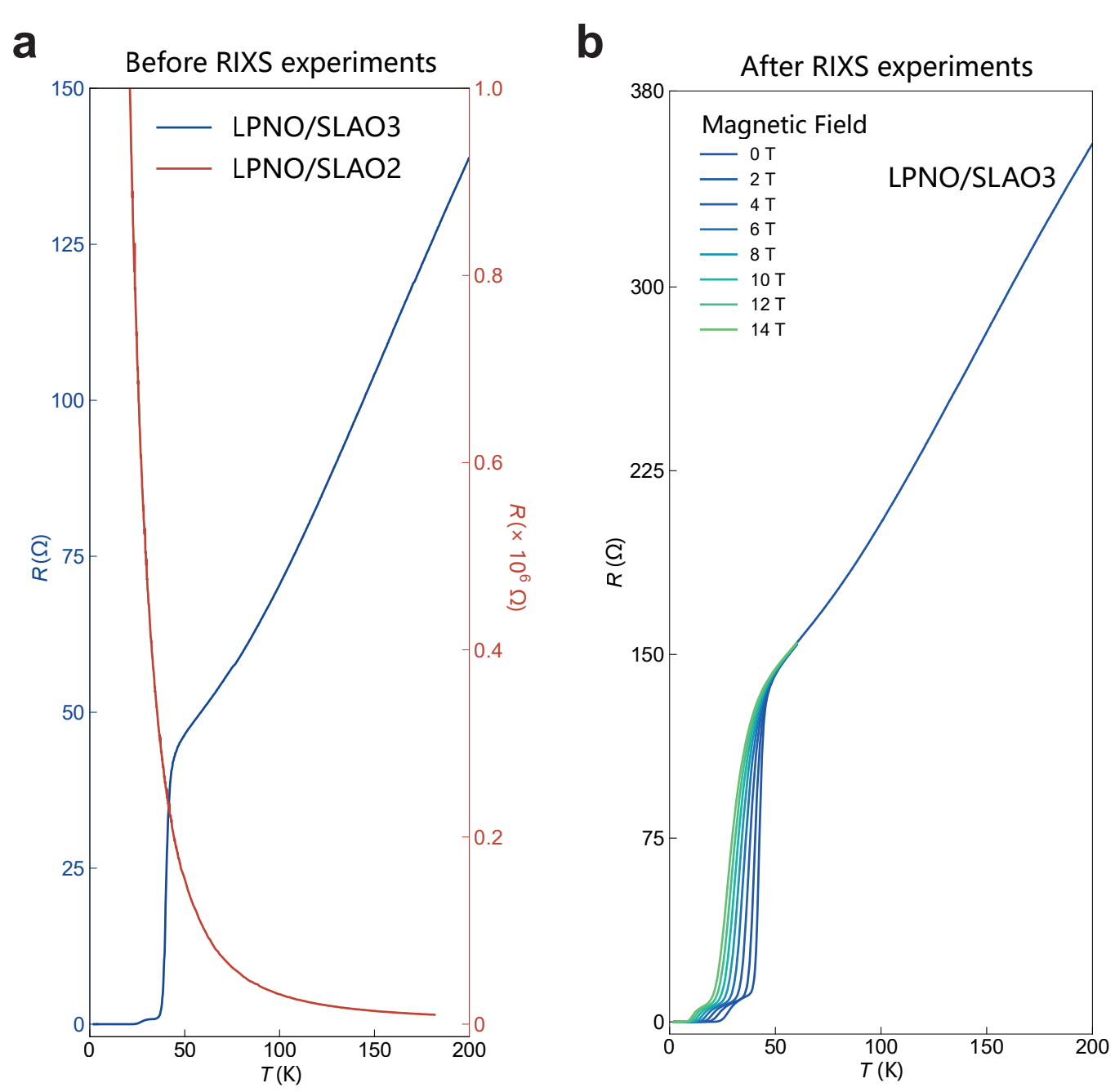

FIG. S2. **Electrical transport of the oxygen-content tuning samples. a** Temperature-dependent resistivity of non-superconducting (non-SC) LPNO/SLAO2 and superconducting (SC) LPNO/SLAO3, measured prior to the RIXS experiments. **b** Temperature-dependent resistivity of SC LPNO/SLAO3 measured after the RIXS experiments. The superconducting transition temperature is suppressed under an applied magnetic field.

Supplementary Note 3. XAS and RIXS experimental geometry.

Figure S3 shows the XAS/RIXS experimental geometry in a top view. During the measurements, the sample was aligned such that the out-of-plane c axis lay in the horizontal scattering plane. The electric field of π-polarized photons lies within the scattering plane, whereas that of σ-polarized photons is perpendicular to the scattering plane. XAS spectra were collected at a grazing incidence angle of $\theta = 20°$ to probe both in-plane and out-of-plane unoccupied states. RIXS measurements were performed with a fixed spectrometer arm angle of $\Omega = 154°$. Incident-energy-dependent RIXS maps were acquired at a grazing incidence angle of $\theta = 20°$, while momentum-dependent RIXS spectra were obtained by rotating the polar angle θ.

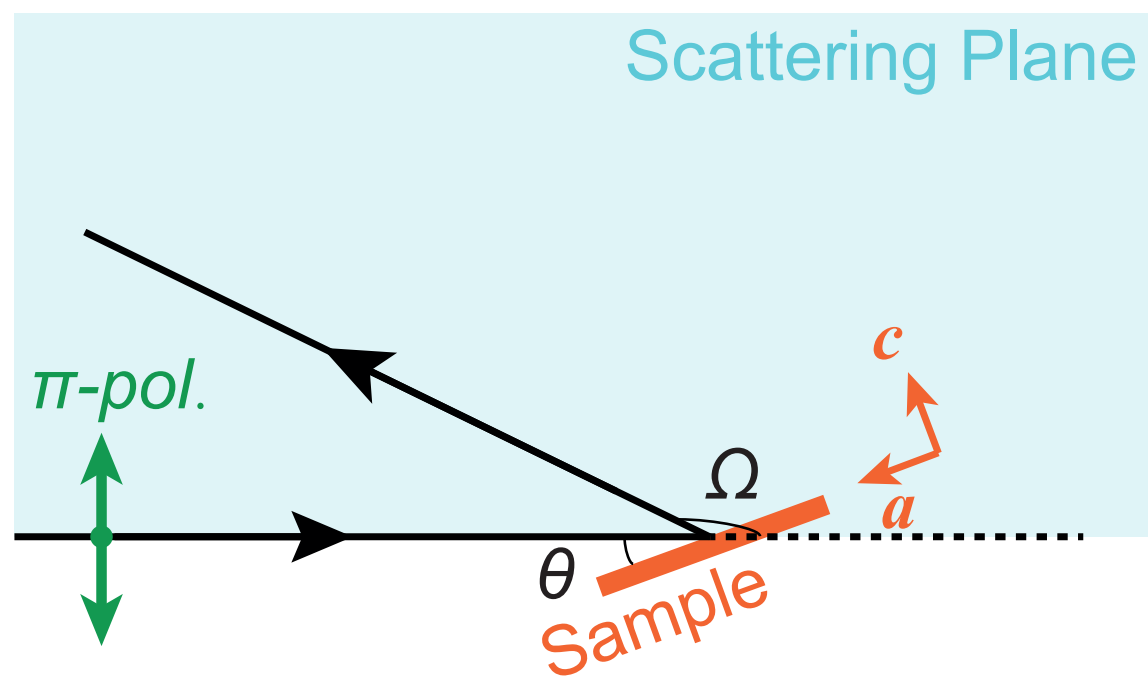

FIG. S3. **XAS and RIXS experimental geometry in a top view.**

Supplementary Note 4. RIXS data analysis and fitting.

All RIXS spectra were first normalized to the incident photon flux and subsequently scaled to the high-energy dd excitations to enable comparison across different samples. The elastic peak position was initially determined from the non-resonant signal of a carbon tape placed near the sample and then refined by fitting the elastic line with a Gaussian or pseudo-Voigt function, using a fixed full width at half maximum (FWHM) corresponding to the instrumental resolution ($\sim$ 40 meV at the Ni L_3 edge).

In the final analysis, a constant background was assumed, and the magnetic excitations were modeled using a damped harmonic oscillator (DHO) response function $\chi''(\omega)$:

$$\chi''(\omega) = \frac{A\gamma\omega}{(\omega^2 - \omega_{ud}^2)^2 + 4\gamma^2\omega^2}.$$

Here, ω is the energy loss, A is the spectral weight prefactor. The ω_{ud} denotes the undamped energy of the magnon, γ represents the damping factor associated with the magnon linewidth. The DHO peak maximum corresponds to the magnon peak energy ω_p. Additional spectral weight at higher energies ($\gtrsim$ 0.1 eV) was accounted for by an extra DHO component with an effective doubled frequency term to capture higher-order magnetic excitations.

None of the RIXS spectra presented in this work have been smoothed. For the two-dimensional RIXS colour maps shown in Figures 2a–2d and Figure 3a, the spectra were linearly interpolated onto a uniform energy-loss grid with a step size of 1 meV. This interpolation interval is substantially smaller than the detector pixel size (~6 meV), ensuring that all experimental features, including the statistical noise, are faithfully preserved without introducing any artificial smoothing.

Figures S4 show representative RIXS spectra of LNO/LAO at $q_\parallel = (-0.06, -0.06)$, together with the corresponding fits. For comparison, the RIXS spectrum of bulk LNO at the same momentum is also overlaid [S6]. A clear difference is immediately observed between the two: while the magnon peak in LNO/LAO is noticeably broader, its peak position remains nearly unchanged. This trend is further confirmed by the fitting results. The DHO analysis yields essentially identical magnon peak energies (ω_p) for both samples (green and orange squares in Fig. S4). In contrast, the stronger damping in LNO/LAO leads to a slightly larger ω_{ud} compared to bulk LNO (green and orange triangles in Fig. S4), although the difference remains within 10 meV. Similar results were reported in Ref. [S7].

In the main text, we present the magnon dispersions of the compressively strained thin films and bulk LNO using ω_p. Here in Fig. S5, we show the corresponding dispersions obtained from ω_{ud}. The magnon bandwidths of the thin films differ only slightly from that of LNO bulk (for example, the difference in ω_{ud} between LNO/LAO and LNO bulk is only ~10 meV). From an experimental perspective, this difference is relatively small. At the Ni L_3 edge, the energy interval between adjacent detector pixels is approximately 6 meV, while the overall experimental energy resolution is about 40 meV. Consequently, a difference of only 10 meV lies within the instrumental uncertainty. In addition, fitting the exchange coupling J using an effective Heisenberg

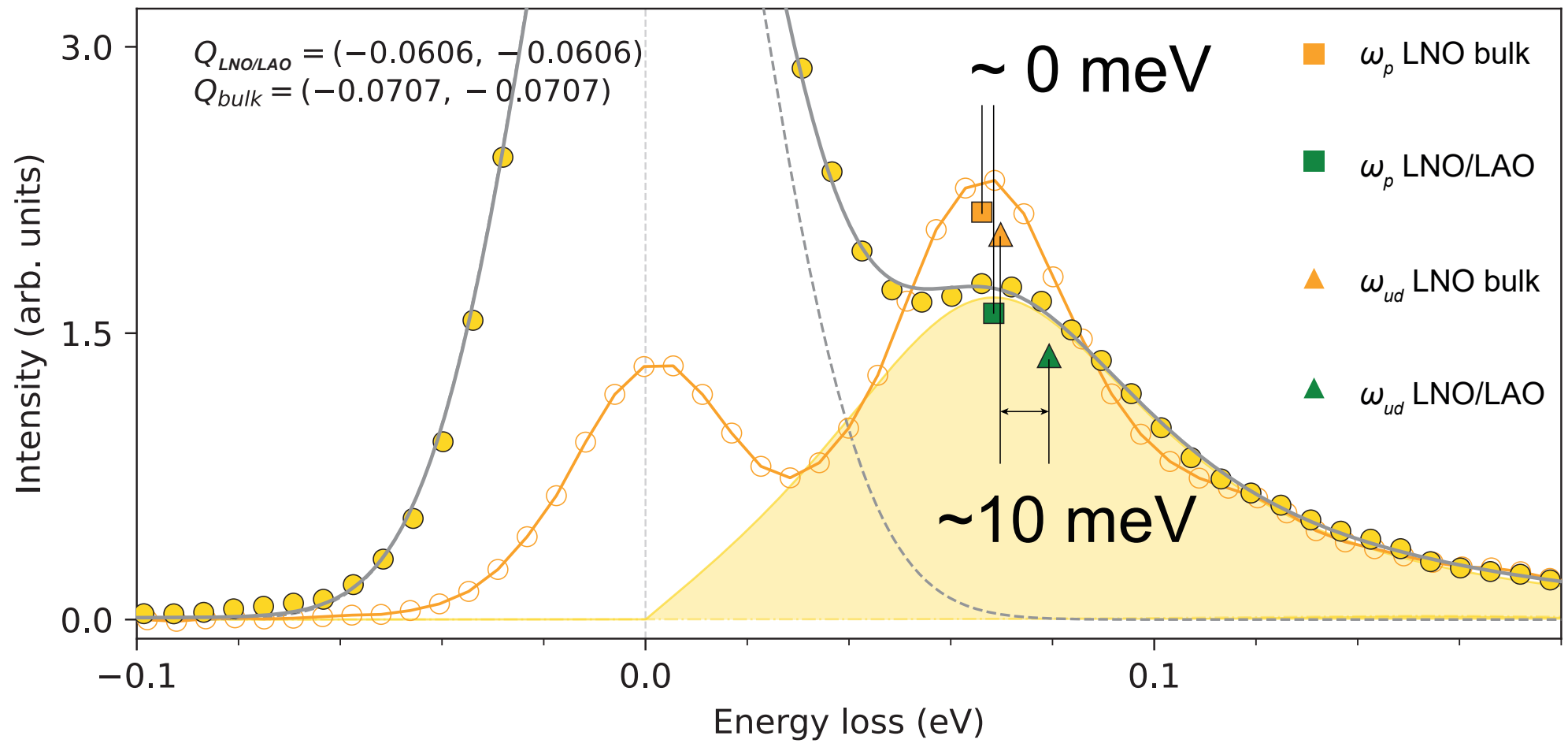

FIG. S4. **Representative DHO fitting of the magnon peak at** $q_{\parallel} = (-0.06, -0.06)$ **for LNO/LAO.** The DHO contribution is shown as the yellow shaded area, and the elastic line is indicated by the dashed curve. The fitted peak positions (ω_p) are marked by green squares (LNO/LAO) and orange squares (bulk LNO), respectively. The corresponding undamped energies (ω_{ud}) are indicated by green triangles (LNO/LAO) and orange triangles (bulk LNO), respectively.

model for such a small difference in the magnon bandwidth is likely to yield values within the fitting error bars. For these reasons, we do not consider the current data sufficient to reliably conclude that the interlayer exchange coupling J_z is enhanced under compressive strain.

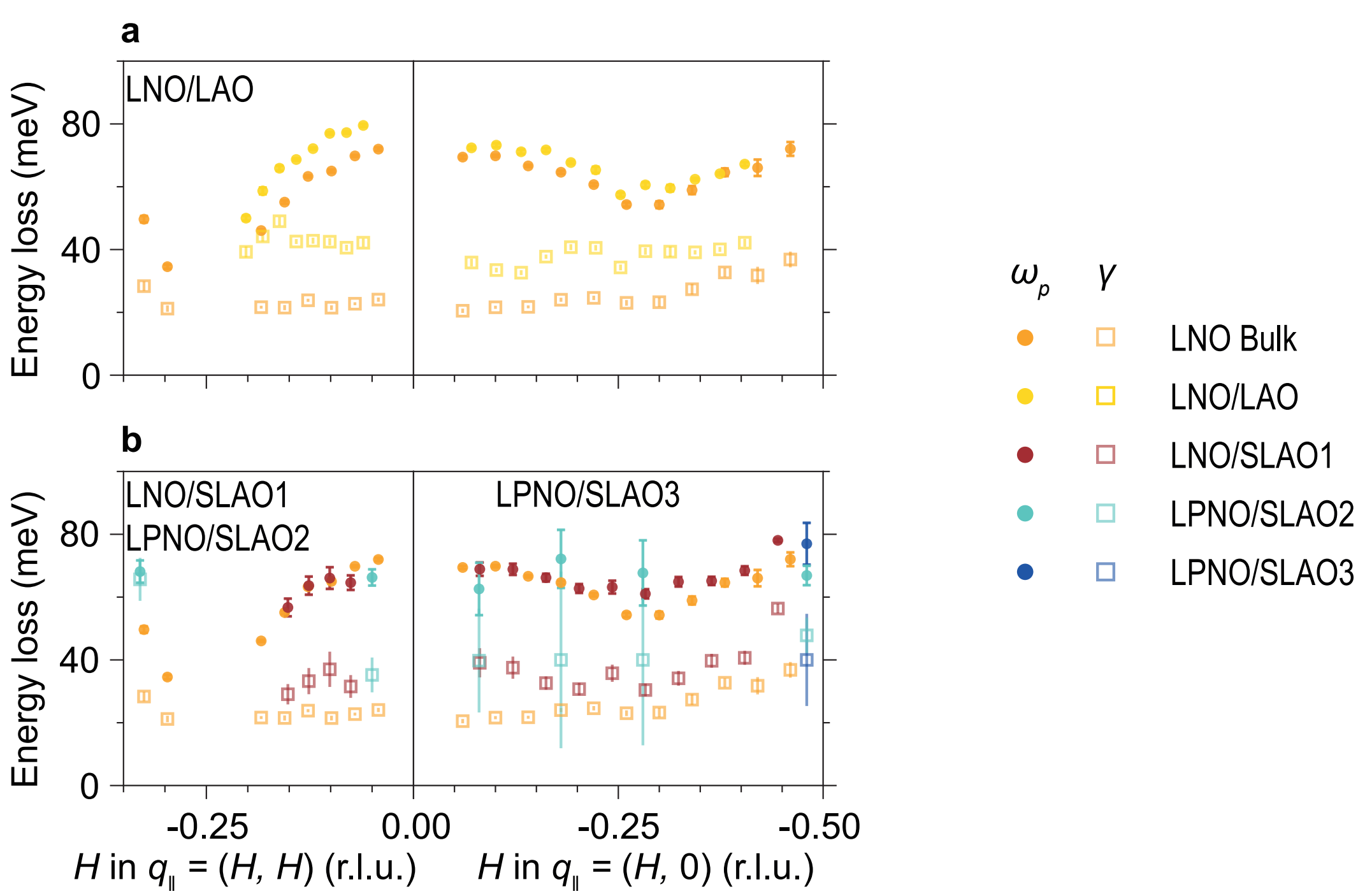

FIG. S5. **Magnon dispersion (**ω_{ud} **vs** $q_{\parallel}$**) of the compressively strained thin films a,** LNO/LAO (yellow circles) **b,** LNO/SLAO1 (brown circles), LPNO/SLAO2 (turquoise circles), and LPNO/SLAO3 (blue circles). The dispersions of LNO bulk (ω_{ud} vs $q_{\parallel}$, orange circles) are overlaid for comparison, which were extracted from Ref. [S6] and processed. The corresponding damping factors (γ) are shown as semi-transparent squares.

Fig. SS6-SS10,The RIXS spectra and corresponding fittings along (H, H) and $(H, 0)$ directions for LNO/LAO, LNO/SLAO1, LPNO/SLAO2 and LPNO/SLAO3.

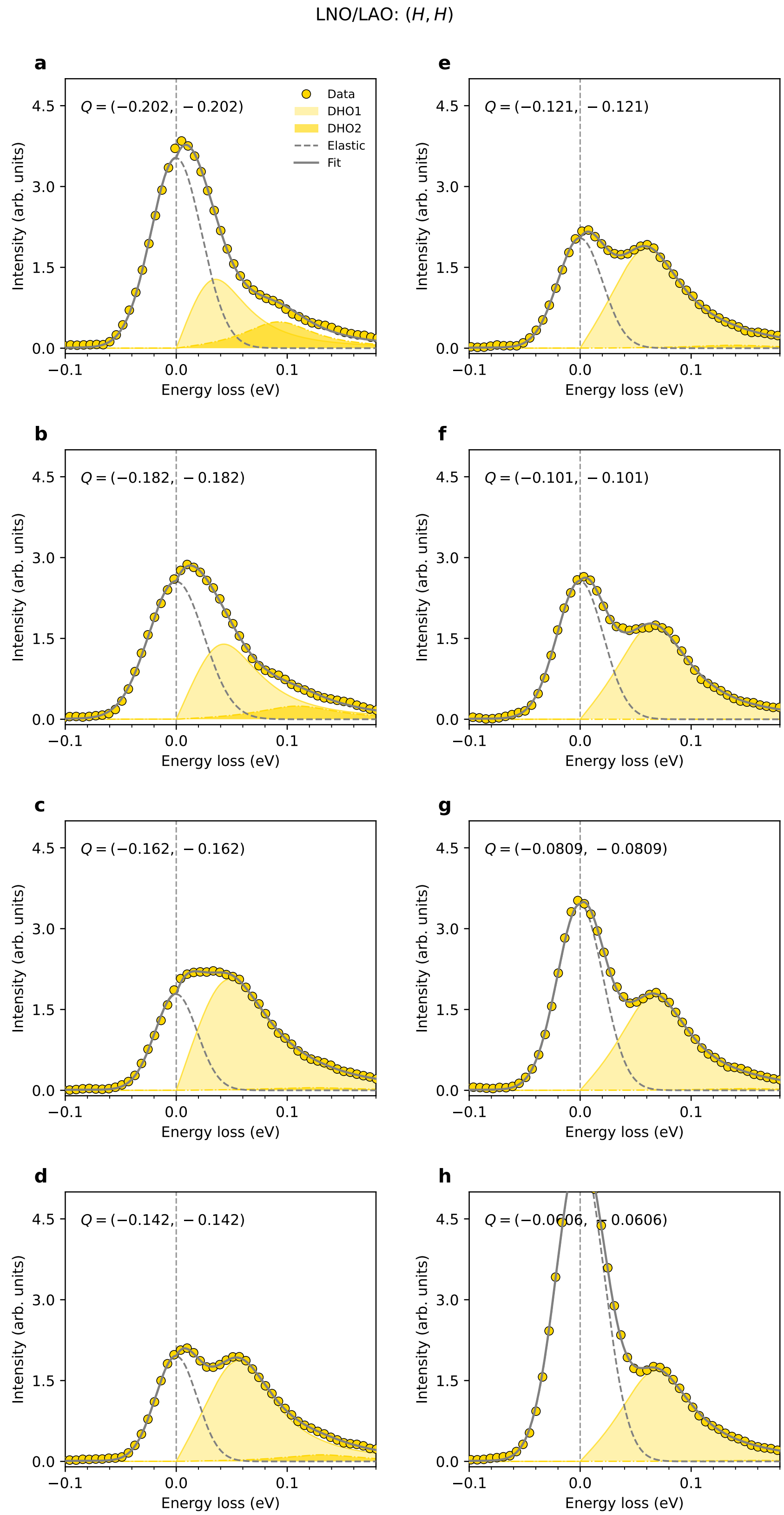

FIG. S6. The raw RIXS spectra and corresponding fitting curves for the LNO/LAO sample measured along the (H, H) direction.

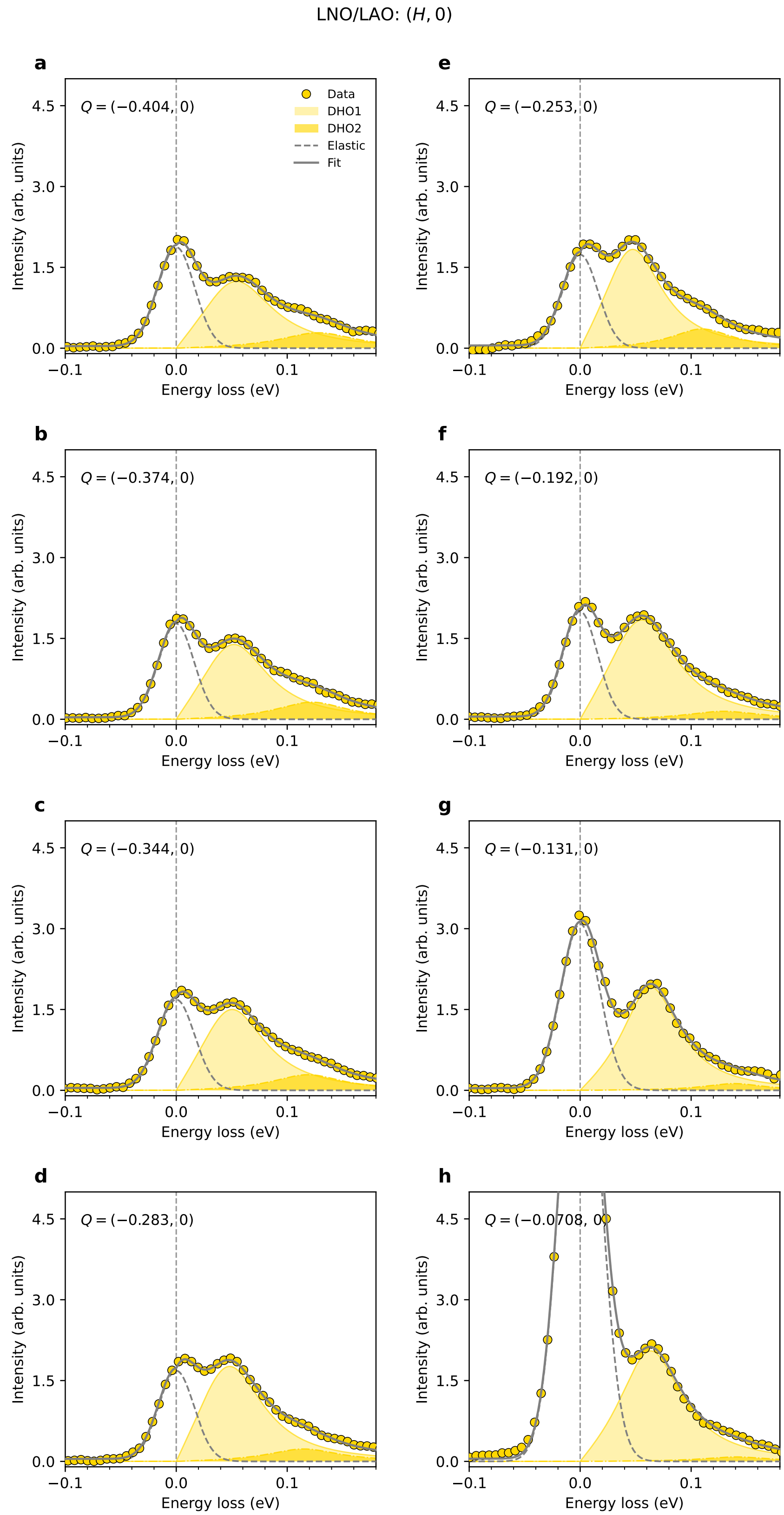

FIG. S7. The raw RIXS spectra and corresponding fitting curves for the LNO/LAO sample measured along the $(H, 0)$ direction.

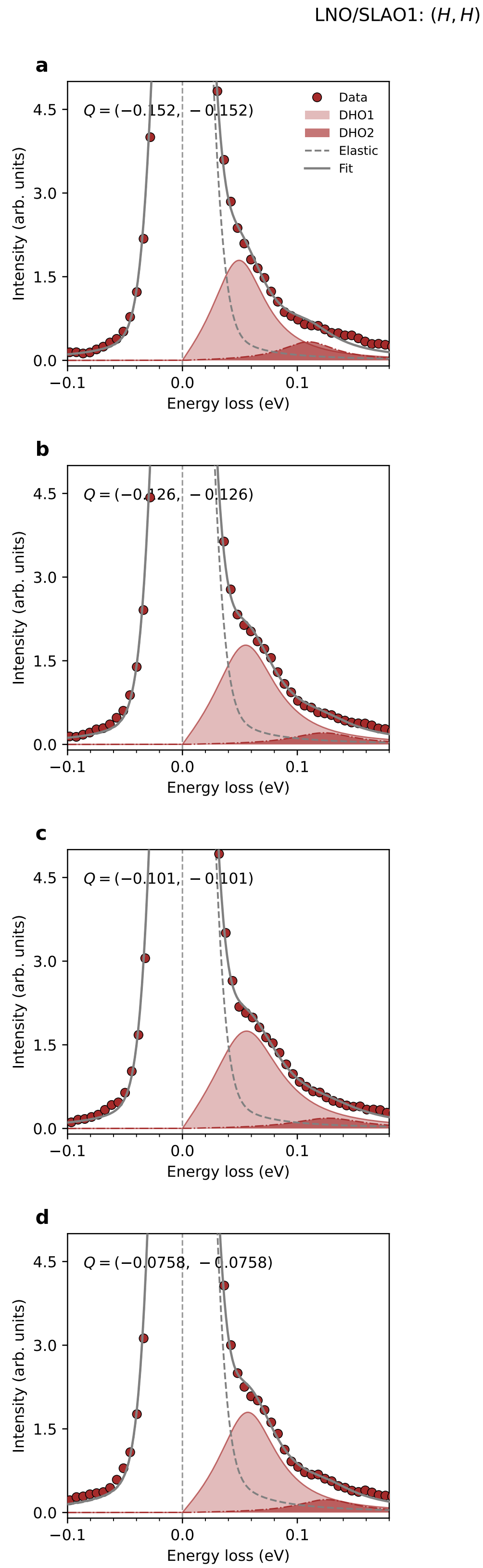

FIG. S8. The raw RIXS spectra and corresponding fitting curves for the LNO/SLAO1 sample measured along the (H, H) direction.

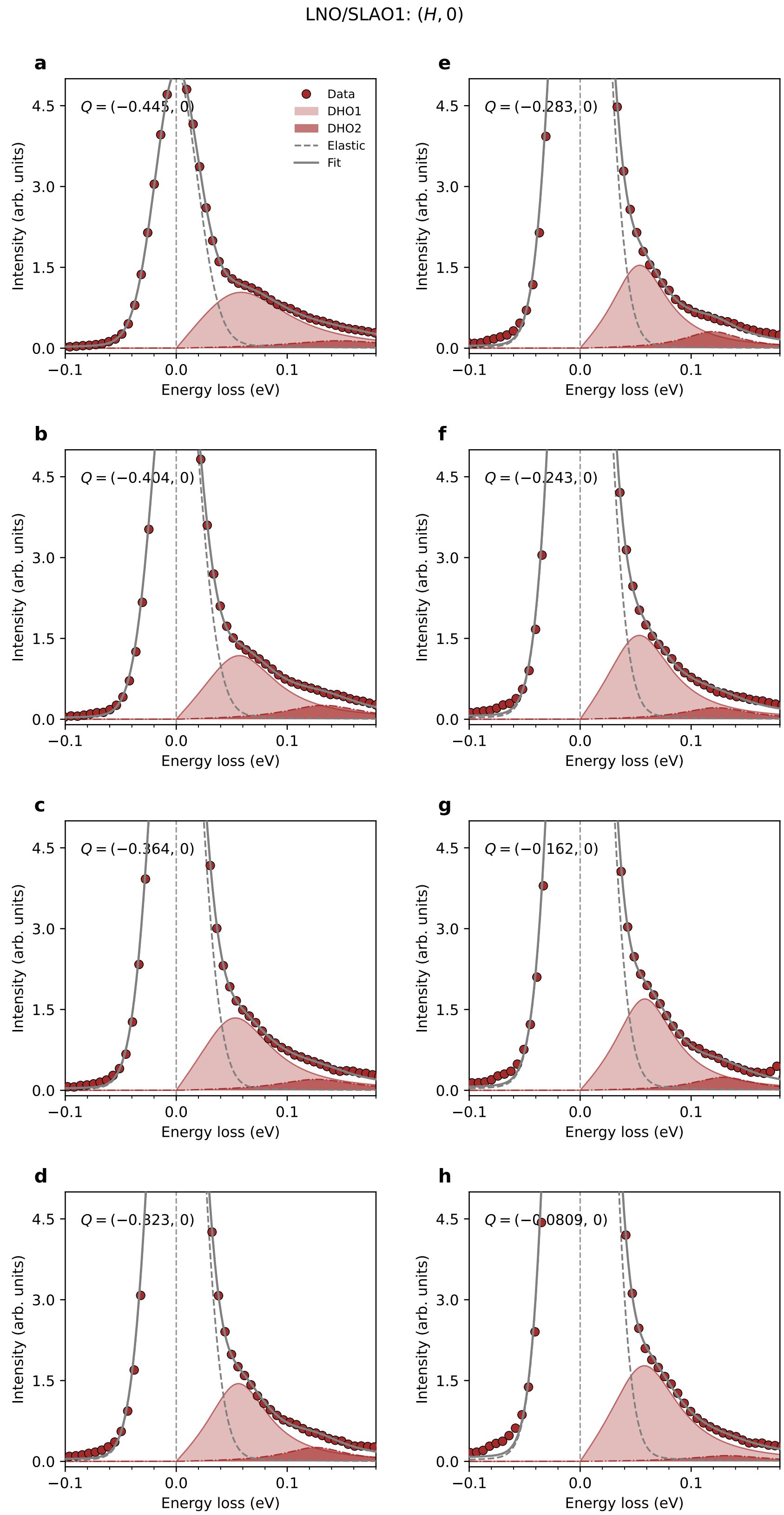

FIG. S9. The raw RIXS spectra and corresponding fitting curves for the LNO/SLAO1 sample measured along the $(H, 0)$ direction.

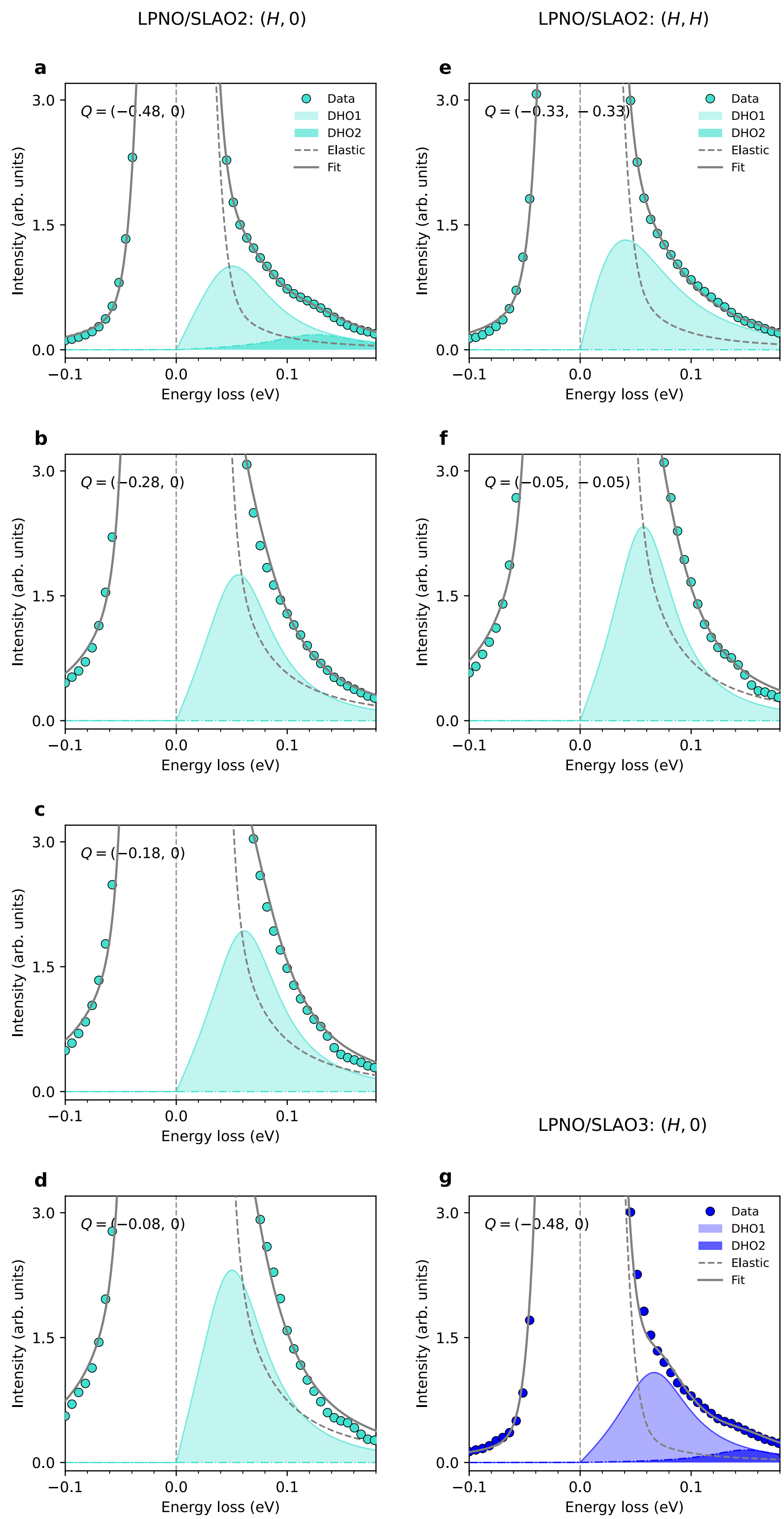

FIG. S10. The raw RIXS spectra and corresponding fitting curves for the LPNO/SLAO2 and LPNO/SLAO3 samples.

Supplementary Note 5. Confirmation of the complete suppression of SDW in the superconducting (La,Pr)$_3$Ni$_2$O$_{7-\delta}$ film.

To confirm the complete suppression of the spin-density-wave (SDW) order in the superconducting LPNO/SLAO3, we first performed a momentum-space scan in the (H, K) plane (Fig. S11) at 53 K, which is above the superconducting transition temperature. No discernible SDW signal is observed, indicating the complete suppression of long-range SDW order in LPNO/SLAO3 is intrinsic.

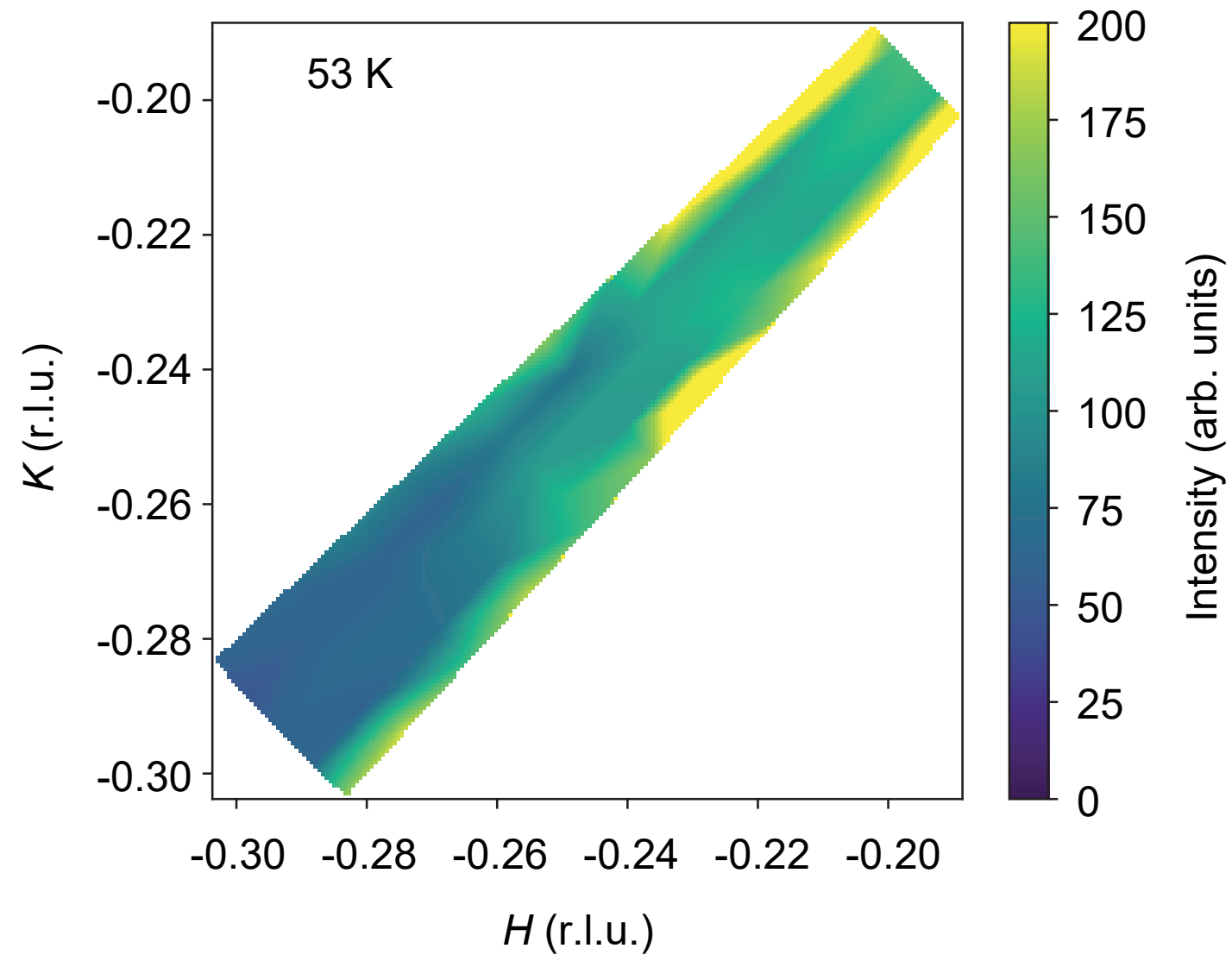

FIG. S11. **(H, K)-space RIXS intensity map of LPNO/SLAO3.** The intensity is integrated over an energy-loss window of $[-0.02, 0.02]$ eV, corresponding to the experimental energy resolution at the Ni L_3 edge, Data were collected at 53 K.

[S1] T. Cui, S. Choi, T. Lin, C. Liu, G. Wang, N. Wang, S. Chen, H. Hong, D. Rong, Q. Wang, *et al.*, Strain-mediated phase crossover in Ruddlesden–Popper nickelates, Communications Materials **5**, 32 (2024).
[S2] L. Bhatt, E. Abarca Morales, A. Y. Jiang, E. K. Ko, Y.-F. Zhao, N. Schnitzer, G. A. Pan, D. Ferenc Segedin, Y. Liu, Y. Yu, *et al.*, Structural modifications in strain-engineered bilayer nickelate thin films, Nature (2026).
[S3] B. Hao, M. Wang, W. Sun, Y. Yang, Z. Mao, S. Yan, H. Sun, H. Zhang, L. Han, Z. Gu, J. Zhou, D. Ji, and Y. Nie, Superconductivity and phase diagram in Sr-doped La$_{3-x}$Sr$_x$Ni$_2$O$_7$ thin films, Nat. Mater. (2025).
[S4] G. Zhou, H. Wang, H. Huang, Y. Chen, F. Peng, W. Lv, Z. Nie, W. Wang, J.-F. Jia, Q.-K. Xue, and Z. Chen, Superconductivity onset above 60 K in ambient-pressure nickelate films, National Science Review , nwag151 (2026).
[S5] Y. Liu, B. Y. Wang, J. Li, Y. Tarn, L. Bhatt, M. Colletta, Y.-M. Wu, C.-T. Kuo, J.-S. Lee, B. H. Goodge, *et al.*, A superconducting half-dome in bilayer nickelates, arXiv preprint arXiv:2603.12196 (2026).
[S6] X. Chen, J. Choi, Z. Jiang, J. Mei, K. Jiang, J. Li, S. Agrestini, M. Garcia-Fernandez, H. Sun, X. Huang, *et al.*, Electronic and magnetic excitations in La$_3$Ni$_2$O$_7$, Nature Communications **15**, 9597 (2024).
[S7] H. Zhong, B. Hao, Z. Zhang, A. Chen, Y. Wei, R. Liu, X. Huang, C. Li, W. Zhang, C. Liu, *et al.*, Spin correlations in La$_3$Ni$_2$O$_7$ superconducting thin films, arXiv preprint arXiv:2502.03178 (2025).
